# Opa1 processing is dispensable in mouse development but is protective in mitochondrial cardiomyopathy

**DOI:** 10.1126/sciadv.adp0443

**Published:** 2024-08-02

**Authors:** Sofia Ahola, Lilli A. Pazurek, Fiona Mayer, Philipp Lampe, Steffen Hermans, Lore Becker, Oana V Amarie, Helmut Fuchs, Valerie Gailus-Durner, Martin Hrabe de Angelis, Dietmar Riedel, Hendrik Nolte, Thomas Langer

**Affiliations:** ^1^ Max Planck Institute for Biology of Ageing, Cologne, Germany.; ^2^ Institute of Experimental Genetics, German Mouse Clinic, Helmholtz Zentrum München, German Research Center for Environmental Health (GmbH), Neuherberg, Germany.; ^3^ Experimental Genetics, TUM School of Life Sciences, Technische Universität München, Freising, Germany.; ^4^ German Center for Diabetes Research (DZD), 85764 Neuherberg.; ^5^ Max Planck Institute for Multidisciplinary Sciences, Göttingen, Germany.; ^6^ Cologne Excellence Cluster on Cellular Stress Responses in Aging-Associated Diseases (CECAD), University of Cologne, Cologne, Germany.

## Abstract

Mitochondrial fusion and fission accompany adaptive responses to stress and altered metabolic demands. Inner membrane fusion and cristae morphogenesis depends on optic atrophy 1 (Opa1), which is expressed in different isoforms and is cleaved from a membrane-bound, long to a soluble, short form. Here, we have analyzed the physiological role of Opa1 isoforms and Opa1 processing by generating mouse lines expressing only one cleavable Opa1 isoform or a non-cleavable variant thereof. Our results show that expression of a single cleavable or non-cleavable Opa1 isoform preserves embryonic development and the health of adult mice. Opa1 processing is dispensable under metabolic and thermal stress but prolongs life span and protects against mitochondrial cardiomyopathy in OXPHOS-deficient *Cox10*^−/−^ mice. Mechanistically, loss of Opa1 processing disturbs the balance between mitochondrial biogenesis and mitophagy, suppressing cardiac hypertrophic growth in *Cox10*^−/−^ hearts. Our results highlight the critical regulatory role of Opa1 processing, mitochondrial dynamics, and metabolism for cardiac hypertrophy.

## Teaser

Inhibition of Opa1 processing in mice allows normal development but exacerbates mitochondrial cardiomyopathy.

## INTRODUCTION

Mitochondria are highly dynamic organelles that constantly fuse and divide. This dynamic behavior of mitochondria ensures mitochondrial inheritance, distribution, and quality control; allows metabolic adaptation of cells; and has been linked to cell death pathways (*1*, *2*). Defects in the mitochondrial fusion or fission machinery are associated with many pathologies, including different encephalomyopathies, cardiomyopathies, and optic atrophies (*3*, *4*).

The dynamin-like guanosine triphosphatase (GTPase) dominant optic atrophy 1 (Opa1) regulates mitochondrial inner membrane (IM) fusion and cristae morphogenesis. *OPA1* mutations cause autosomal dominant optic atrophy (ADOA) in humans, which is associated with the selective loss of retinal ganglion cells and blindness (*5*, *6*). Missense mutations in *OPA1 *lead to multisystem disorders with a wide range of clinical manifestations (*7*). Numerous studies in mice have demonstrated the critical role of Opa1 in mitochondrial function. Impaired fusion and cristae morphogenesis in *Opa1^−/−^* mice results in embryonic lethality, while tissue-specific knockout models in energy-demanding tissues such as the brain, heart, and skeletal muscle are associated with respiratory defects and cause organ dysfunction and animal death (*8*–*14*). In contrast, liver-specific loss of Opa1 impairs cristae morphogenesis but does not affect organ function due to a mitohormetic stress response (*15*), which protects against drug-induced liver injury and nonalcoholic fatty liver disease (*16*). On the other hand, a protective effect of mild overexpression of Opa1 has been observed in mouse models for oxidative phosphorylation (OXPHOS) dysfunction (*12*, *17*, *18*), demonstrating the importance of Opa1 levels for physiological functions.

Opa1 function is tightly regulated at the protein level. Following import of nuclear-encoded Opa1 into mitochondria and removal of the mitochondrial targeting sequence, the maintenance of normal mitochondrial morphology depends on the proteolytic processing of Opa1 and the balanced accumulation of membrane-bound, uncleaved (long, l-Opa1) and soluble, cleaved (short, s-Opa1) forms (*19*). The IM proteases overlapping activity with m-AAA protease 1 (Oma1) and Yeast mitochondrial escape protein 1-like (Yme1l) cleave l-Opa1 at neighboring proteolytic sites S1 and S2, respectively, to generate s-Opa1 forms (*20*–*25*). Yme1l also mediates Opa1 turnover, thereby limiting the overall Opa1 accumulation (*26*). Studies in cultured cells and reconstituted systems have shown that l-Opa1 is essential and, together with cardiolipin, sufficient to mediate membrane fusion (*20*, *27*, *28*), which, however, is further stimulated in the presence of s-Opa1 (*28*, *29*). Under basal conditions, both Yme1l and Oma1 mediate limited processing of Opa1 (*23*, *25*). Mitochondrial stress, such as dissipation of the membrane potential, oxidative, or thermal stress, further activates Oma1, which converts l-Opa1 into s-Opa1 and promotes mitochondrial fragmentation (*21*, *24*, *30*). Similar to l-Opa1, s-Opa1 is sufficient to maintain cristae morphogenesis and respiration when expressed in *Opa1*^−/−^ mouse embryonic fibroblasts (MEFs) (*27*, *31*). s-Opa1 associates with the membrane, induces tubulation by forming helical assemblies, and compacts the membrane via dimerization of the GTPase domain upon guanosine 5′-triphosphate binding (*32*, *33*). Accordingly, l-Opa1 has been proposed to mark the sites for fusion, which may explain the increased fusion efficiency with balanced accumulation of l- and s-Opa1 (*29*, *32*).

**Fig. 1.**
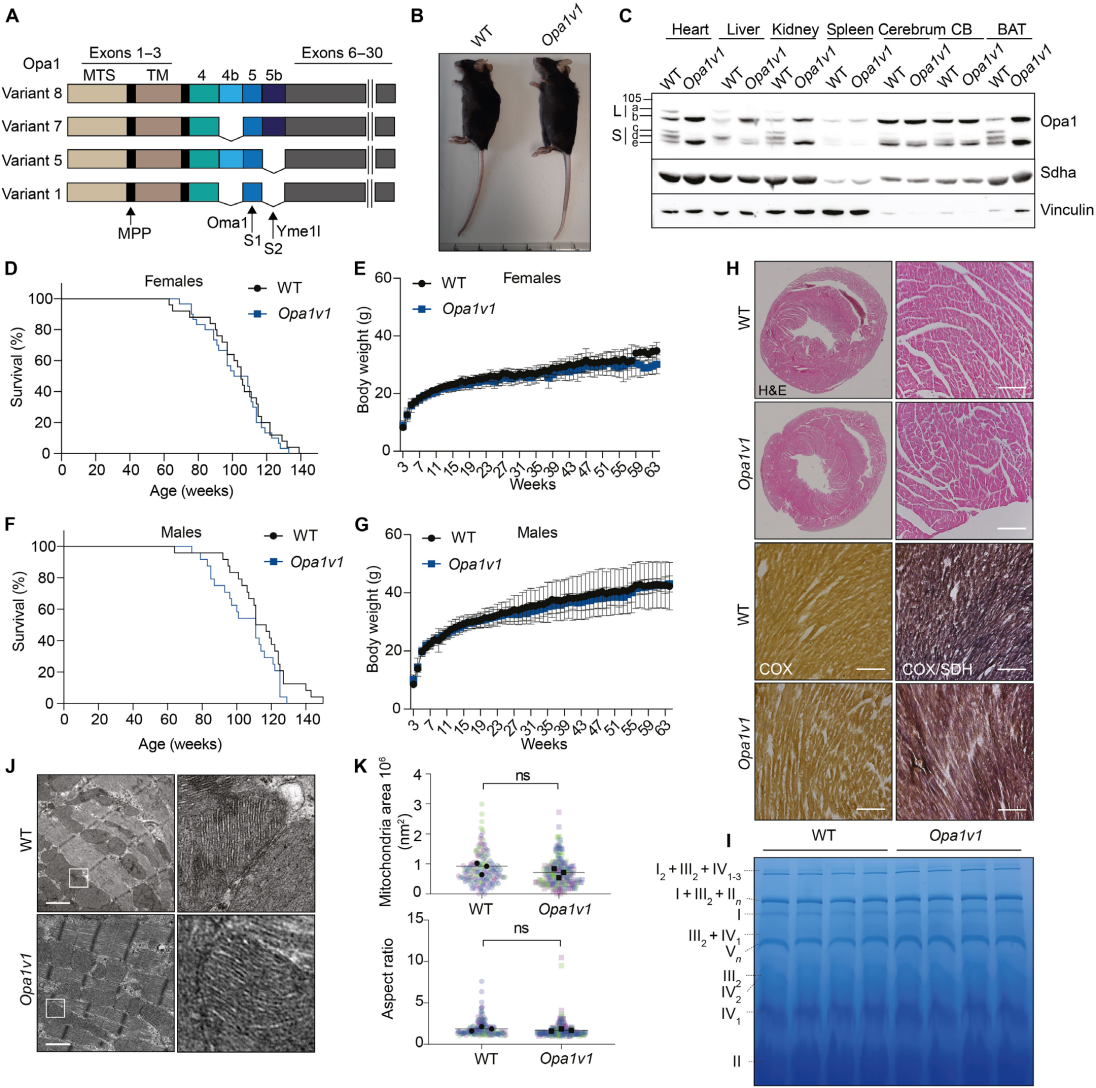
Expression of only Opa1 V1 sustains mouse development and mitochondrial structure. (**A**) Illustration of *Opa1* gene variants showing all four isoforms expressed in mice. Positions of processing sites S1 and S2 for Oma1 and Yme1l proteases, respectively, are indicated. (**B**) Twelve-week-old knock-in mice expressing only *Opa1v1* develop normally and show no obvious phenotype. (**C**) V1 expression in tissues of wild-type (WT) and *Opa1v1* mice. (**D** to **G**) *Opa1v1* female and male mice have a normal life span (WT, male, *n* = 26; WT, female, *n* = 25; *Opa1v1*, male, *n* = 25; *Opa1v1*, female, *n* = 30) and show similar body weight gain as their WT littermates (*n* = 10). (**H**) Normal heart structure and normal COX/SDH activity in 12-week-old WT and *Opa1v1* mice, as shown by hematoxylin and eosin (H&E) or COX/SDH staining, respectively. Scale bar, 200 μm. (**I**) Blue native (BN)–polyacrylamide gel electrophoresis (PAGE) from 56-week-old WT and *Opa1v1* heart mitochondria showing RCC and super complexes (*n* = 4). (**J**) Representative transmission electron microscopy (TEM) images from 12-week-old WT and *Opa1v1* heart tissue showing normal mitochondrial morphology and cristae structure. Scale bars, 1 μm. (**K**) Mitochondrial area and aspect ratio in hearts (*n* = 3 mice; mitochondria WT, *n* = 181; and *Opa1v1*, *n* = 231 in total). MTS, mitochondrial targeting sequence; TM, transmembrane domain; MPP, Mitochondrial-processing peptidase processing site; CB, cerebellum; BAT, brown adipose tissue; ns, nonsignificant.

Opa1 is expressed in eight different mRNA isoforms in humans and four in mice, which are produced by alternative splicing and which are differentially expressed in different organs (*34*). The *Opa1* isoforms differ only in the presence of exons 4, 4b, and 5b, the latter encoding the proteolytic processing site S2 for Yme1l (Fig. 1A). In contrast, all expressed human and mouse splice variants contain the Oma1 processing site S1, pointing to important functions of Oma1-mediated Opa1 processing.

The role of Opa1 processing and s-Opa1 in vivo has been mainly studied using Oma1- or Yme1l-deficient mouse models (*35*–*39*). However, the identification of an increasing number of substrates of both peptidases in addition to Opa1 has complicated the interpretation of these models (*26*, *40*–*44*). The physiological role of the different splice variants in vivo is now unknown. Selective silencing of the three isoform-specific exons in HeLa cells revealed different effects on mitochondrial morphology and bioenergetics, suggesting isoform-specific functions (*45*). Similarly, only co-expression of two isoforms of Opa1 in *Opa1*^−/−^ MEFs allowed complete recovery of the mitochondrial morphology (*31*).

To analyze the physiological role of Opa1 processing and different *Opa1* mRNA isoforms, we generated two knock-in mouse models by CRISPR-Cas9–mediated genome editing of the *Opa1* locus. One mouse line expresses exclusively Opa1 variant 1 (V1), which lacks exons 4b and 5b and thus only contains the Oma1 processing site S1 (*Opa1v1*). The second mouse line expresses a mutant form of Opa1 V1, which lacks four amino acids at the S1 cleavage site inhibiting Oma1 cleavage (*Opa1v1*Δ*4*). These *Opa1* mouse models provide insight into the role Opa1 splice variants and Opa1 processing in vivo.

## RESULTS

### Mouse embryonic development and viability are maintained by expression of only Opa1 V1

Four Opa1 isoforms are expressed in mice, which differ in the presence or absence of cleavage sites for Oma1 (S1 within exon 5) and for Yme1l (S2 within exon 5b) (Fig. 1A). To allow the analysis of Oma1-mediated Opa1 processing without the potential confounding effects of Yme1l cleavage, we used CRISPR-mediated genome editing to generate transgenic *Opa1* knock-in mice that exclusively express Opa1 V1 (fig. S1A and table S1). V1 harbors the Oma1 cleavage site S1 at arginine 194 but lacks exons 4b and 5b and the Yme1l cleavage sites S3 and S2, respectively (*46*). To exclude potential off-target effects on the phenotype, we bred two genetically independent lines from F_O_ heterozygous pups and backcrossed them for >10 generations. Targeted locus amplification (TLA) sequencing confirmed the integration of the repair nucleotide into the *Opa1* locus on chromosome 16 and did not reveal off-target integrations.

While deletion of *Opa1* in mice is embryonic lethal (*9*, *10*), homozygous *Opa1v1* mice were born healthy at the expected Mendelian ratio (fig. S1B) and showed no apparent phenotype (Fig. 1B). *Opa1v1* was ubiquitously expressed in various tissues of the mice. As expected, we detected only one l-Opa1 form (non-cleaved, mature V1) and one s-Opa1 form (corresponding to Opa1 form e generated by Oma1-mediated processing of V1) (Fig. 1C). Total Opa1 levels were similar in WT and *Opa1v1* hearts (fig. S1C). Opa1 form d generated by Yme1l cleavage was not produced in any tissue (Fig. 1C). Similar Opa1 forms accumulated in wild-type (WT) and *Opa1v1* brain tissues, which is consistent with the predominant expression of Opa1 isoform 1 in the mouse brain (*47*). However, different Opa1 forms accumulated in *Opa1v1* mice compared to those in WT mice in tissues that express several Opa1 isoforms, such as the heart, brown adipose tissue (BAT), and kidney (Fig. 1C). In the kidney and the brain, we detected low amounts of a small from of Opa1, which may represent an aberrant proteolytic cleavage in the absence of S2 and S3 or another posttranslational modification (Fig. 1C). Survival and body weight of both of the sexes were similar in WT and *Opa1v1* littermates (Fig. 1, D to G). Heart ultrastructure was unaffected in *Opa1v1* mice (Fig. 1H), and histological analysis of cardiac tissue did not reveal any OXPHOS deficiency (Fig. 1H). Blue native (BN)–polyacrylamide gel electrophoresis (PAGE) from cardiac mitochondria showed normal respiratory chain complex (RCC) assembly (Fig. 1I). We observed normal mitochondrial size and an unaltered cristae structure by transmission electron microscopy (TEM) (Fig. 1, J and K). These results demonstrate that the expression of V1 is sufficient for normal mouse development and maintains normal mitochondrial morphology and cristae morphogenesis in the heart in vivo.

### Cleavage site mutations inhibit Oma1-mediated V1 processing

**Fig. 2.**
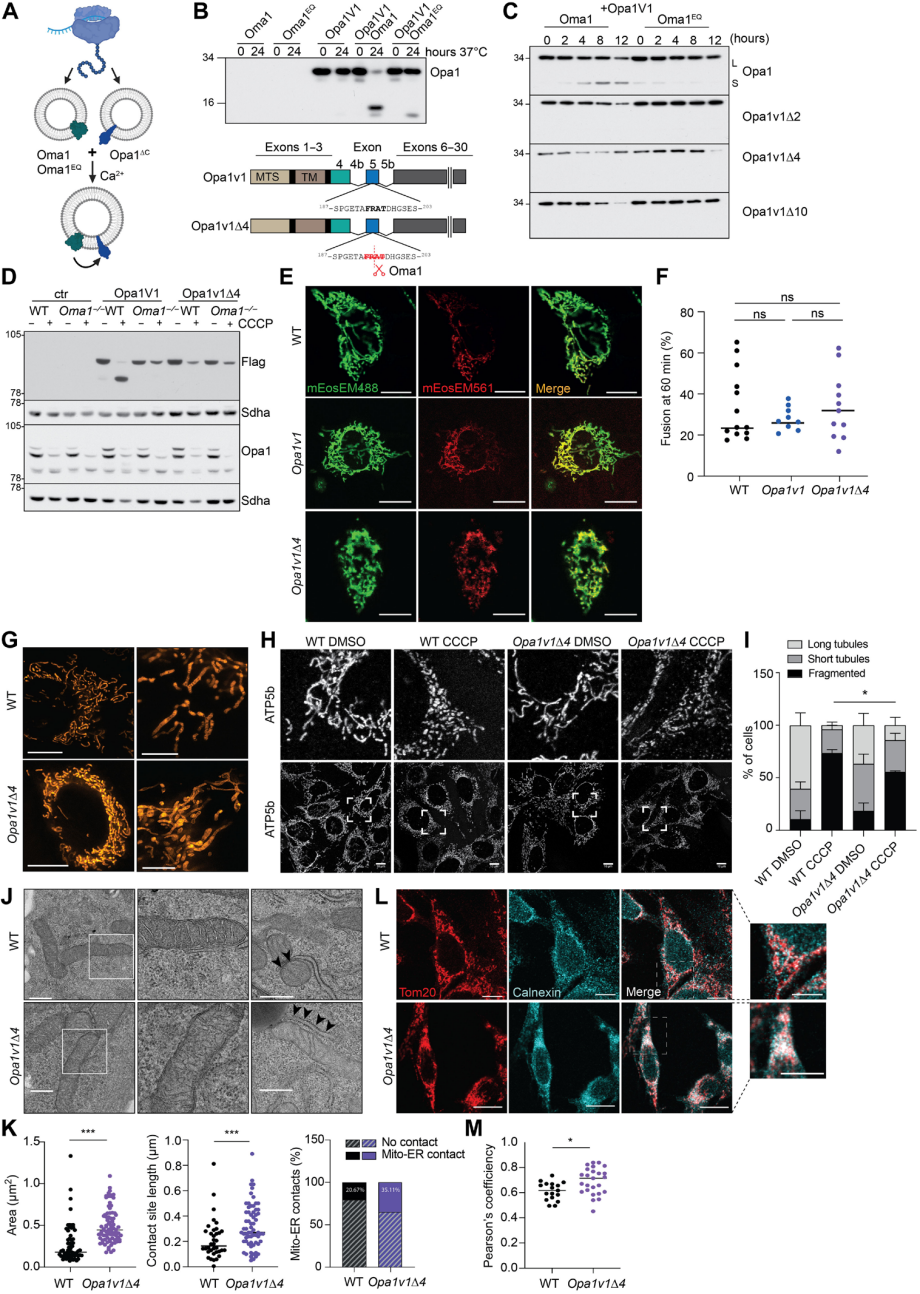
Non-cleavable Opa1 V1 supports mitochondrial fusion in vitro. (**A**) Cell-free assay to monitor Opa1 cleavage by Oma1. Scheme was created with BioRender.com. (**B**) C-terminally truncated Opa1 variant V1 (V1ΔC), Oma1, or Oma1^E324Q^ was synthesized in a cell-free system with liposomes and monitored by SDS-PAGE. V1ΔC is processed by Oma1 but not Oma1^E324Q^ in vitro. (**C**) Deletion of 2 (Δ2), 4 (Δ4), or 10 (Δ10) amino acids at S1 from Opa1v1ΔC blocks Oma1-mediated processing. (**D**) Opa1 processing in WT and *Oma1^−/−^* MEFs transiently expressing Flag-tagged Opa1 variants ± CCCP (2 hours, 20 μM). (**E**) Representative images of WT, *Opa1v1*, and *Opa1v1*Δ*4* MEFs transiently expressing mito-mEosEM488 and mito-mEosEM561 60 min after photoactivation. Scale bars, 10 μm. (**F**) Quantification of fused mitochondrial area in WT (*n* = 13), *Opa1v1* (*n* = 9), and *Opa1v1*Δ*4* (*n* = 11) cells. (**G**) Mitochondrial morphology in WT and *Opa1v1*Δ*4* MEFs. Mitochondria stained with MitoOrange and analyzed by STED nanoscopy. Scale bars, 10 μm and (zoomed in) 5 μm. (**H** and **I**) Immunofluorescence analyses of WT and *Opa1v1*Δ*4* MEFs with ATP5b-specific antibodies ± CCCP (2 hours, 20 μM). Scale bars, 10 μm. Mitochondria of at least 100 cells were quantified per condition in *n* = 3 independent biological replicates and categorized in long tubules, short tubules, and fragmented. *P < 0.05 [two-way analysis of variance (ANOVA)]. (**J** and **K**) Mitochondrial ultrastructure in WT and *Opa1v1*Δ*4* MEFs analyzed by TEM. Scale bars, 500 nm. Mitochondrial size quantification (three independent experiments; mitochondria WT, *n* = 122; and *Opa1v1*Δ*4*, *n* = 214 in total) and mitochondria-ER contact sites (three independent experiments; WT, *n* = 179; and *Opa1v1*Δ*4*, *n* = 188 total contact sites), ***P < 0.001 (unpaired *t* test). (**L** and **M**) Immunofluorescence analyses of WT and *Opa1v1*Δ*4* MEFs with Tom20- and Calnexin-specific antibodies. Colocalization of ER and mitochondria indicated by white color is more prominent in *Opa1v1*Δ*4* MEFs (inset). Scale bars, 10 μm (inset, 20 μm). *P < 0.05.

To investigate the physiological role of Opa1 processing in vivo, we aimed to generate mice expressing only a non-cleavable form of V1. To identify mutations in V1 that would disrupt Oma1 cleavage, we used a cell-free expression system to reconstitute Oma1 and proteolytically inactive Oma1^E324Q^, which has a point mutation in the proteolytic site, in liposomes in vitro (Fig. 2A). After co-expression of V1, we observed cleavage of V1 to Opa1 form e by Oma1 but not by Oma1^E324Q^ (Fig. 2B). We then deleted 2, 4, or 10 amino acids at the Oma1 processing site between Arg^194^ and Ala^195^ of V1 and monitored its cleavage by Oma1 in vitro. Deletion of Arg^194^ and Ala^195^ was sufficient to completely inhibit V1 cleavage (Fig. 2C). Similarly, when expressed in WT or *Oma1*^−/−^ MEFs, we did not observe specific proteolytic processing of V1 variants lacking two or more amino acids at S1 (fig. S1D). Deletion of only Arg^194^ alone did not inhibit Oma1-mediated cleavage of V1 (fig. S1D). As we observed unspecific degradation of V1 variants missing one or two amino acids in these experiments, we focused on V1 missing four amino acids (V1Δ4). V1Δ4 was protected from proteolytic processing by Oma1, even upon dissipation of the mitochondrial membrane potential (ΔΨ_m_), which is known to activate Oma1 (Fig. 2D) (*21*). The steady-state level of V1Δ4 was decreased in depolarized mitochondria, likely reflecting increased turnover by Yme1l.

To test whether V1Δ4 is functional and mediates mitochondrial fusion, we used MEFs expressing the mitochondrially targeted photoconvertible red fluorescent protein mito-mEosEM561, V1, and V1Δ4 from the genomic *Opa1* locus. Mito-mEosEM561 was locally activated, and the distribution of activated mEosEM was monitored by time-lapse confocal imaging (Fig. 2E). We observed similar fusion rates in V1 and V1Δ4 expressing cells as in the WT, indicating that V1 as well as non-cleavable V1Δ4 preserves mitochondrial fusion (Fig. 2F). Consistently, V1Δ4 cells contained tubular mitochondria with largely normal cristae morphology, although mitochondria were more clustered around the nucleus (Fig. 2G). Depolarization of mitochondria caused mitochondrial fragmentation in WT and V1Δ4 cells (Fig. 2, H and I). To exclude that the mitochondrial clustering is caused by cell shrinkage, we analyzed the cell diameter by fluorescence activated cell sorting (FACS) and found similar sizes of WT and V1Δ4 cells (fig. S1H). V1Δ4 cells showed slightly enlarged mitochondria, when analyzing the mitochondrial ultrastructure by TEM (Fig. 2, J and K). These observations are consistent with previous studies demonstrating that l-Opa1 is sufficient to largely maintain mitochondrial fusion, which, however, can be stimulated by s-Opa1 (*28*, *29*). TEM images from V1Δ4 expressing cells showed increased endoplasmic reticulum (ER)–mitochondrial contact sites (Fig. 2, J and K). ER has been shown to regulate mitochondrial dynamics by marking and possibly constricting the fission sites (*48*). Co-staining of mitochondria and ER showed increased co-localization in V1Δ4 cells compared to that in WT cells (Fig. 2, L and M). Together, loss of Opa1 processing mildly impairs mitochondrial distribution and prolongs ER-mitochondrial contact sites.

### Opa1 processing is dispensable for normal mouse development and health

**Fig. 3.**
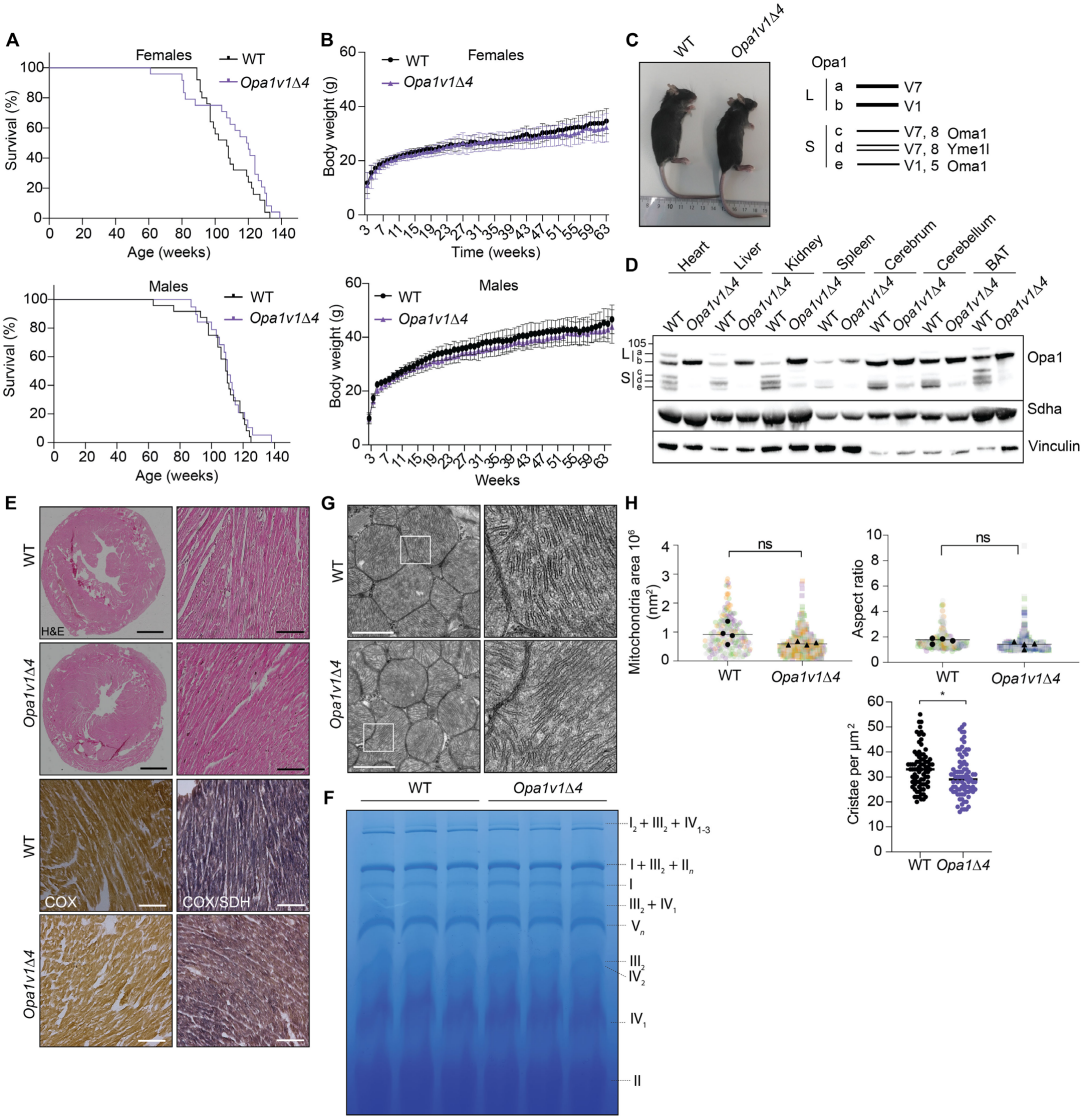
Expression of V1Δ4 allows normal mouse development and preserves mitochondrial structure in vivo. (**A**) Life span of WT and *Opa1v1*Δ*4* mice (WT, male, *n* = 25; WT, female, *n* = 30; *Opa1v1*Δ*4*, male, *n* = 24; and *Opa1v*Δ*41*, female, *n* = 30). (**B**) Body weight gain of WT (*n* = 10) and *Opa1v1*Δ*4* mice (*n* = 10) mice. (**C**) Normal development of *Opa1v1*Δ*4* mice. (**D**) Opa1v1Δ4 is expressed throughout mouse tissues and mostly accumulates as mature, non-cleaved l-Opa1. Steady-state levels of succinate dehydrogenase (SDH) subunit A (Sdha) and vinculin were monitored as controls. (**E**) Normal heart structure and normal COX/SDH activity in 12-week-old WT and *Opa1v1*Δ*4* mice, as shown by H&E or COX/SDH staining, respectively. Scale bars, 200 μm. (**F**) BN-PAGE from 56-week-old WT and *Opa1v1*Δ*4* heart mitochondria showing RCC and respiratory supercomplexes (*n* = 3). (**G**) Representative TEM images from 12-week-old WT and *Opa1v1*Δ*4* heart tissue showing normal mitochondrial morphology and cristae structure. Scale bars, 100 nm. (**H**) Quantification of mitochondrial area and aspect ratio in hearts (*n* = 3 mice; mitochondria WT, *n* = 247; and *Opa1v1*, *n* = 405 in total; measurements from different mice are color-coded). Cristae density was calculated for 80 mitochondria from each *n* = 3 animals in WT and *Opa1v1*Δ*4*. The number of cristae was normalized to the mitochondrial area.

To analyze the role of Opa1 processing and s-Opa1 in vivo, we generated *Opa1v1*Δ*4* mice using a strategy similar to that used to generate *Opa1v1* mice but with a repair oligonucleotide encoding V1Δ4 (fig. S1F). We detected no off-target integration by TLA sequencing in an *Opa1v1*Δ*4* pup born after pronuclear injection of guide RNAs (gRNAs) and Cas9. Heterozygous intercrosses produced homozygous *Opa1v1*Δ*4* animals at the expected Mendelian ratio (fig. S1G). *Opa1v1*Δ*4* mice developed normally, had a normal life span, gained body weight as WT mice, and showed no apparent phenotype (Fig. 3, A to C). We next analyzed Opa1 processing in different tissues from 12-week-old WT and *Opa1v1*Δ*4* animals. Oma1-mediated Opa1 processing was severely impaired in all tissues and almost completely blunted in the heart, liver, and BAT, which normally express variants 1 and 7 that can be processed by Yme1l (Fig. 3D) (*47*). Low levels of a short form of Opa1 were detected in the brain and the kidney (Fig. 3D), which may reflect increased Oma1 activity in these tissues. Therefore, we focused our further experiments on the heart. Heart size and structure and mitochondrial OXPHOS function appeared normal in *Opa1v1*Δ*4* animals (Fig. 3, E and F, and fig. S1E). Analysis of heart tissue by TEM revealed normal mitochondrial size and ultrastructure in *Opa1v1*Δ*4* mice, with mild reduction in cristae number in *Opa1v1*Δ*4* hearts, suggesting that s-Opa1 accumulation is dispensable for cristae morphogenesis in the heart in vivo (Fig. 3, G and H). Consistent with this, we have previously observed normal mitochondrial ultrastructure in mice lacking the Opa1 processing peptidases Oma1 and Yme1l in cardiomyocytes (*35*). We conclude from these experiments that V1Δ4 is sufficient to maintain normal development and animal health and that mitochondrial function and cristae formation in the heart is largely unaffected by the loss of s-Opa1 in vivo.

Mutations in the *OPA1* gene in humans most commonly lead to ADOA with loss of vision combined with neuromuscular multisystemic dysfunctions such as ataxia and peripheral neuropathy (*5*, *6*). To investigate whether *Opa1v1* or *Opa1v1*Δ*4* animals show sensory defects, muscle weakness, or motor dysfunction, we performed grip strength, rotarod, and hotplate tests on 1-year-old mice. *Opa1v1* females and *Opa1v1*Δ*4* males showed mild reduction in four paw grip strength tests (fig. S2, A and B), but all the other analyses showed no differences between the genotypes (fig. S2, C and D). To investigate the occurrence of optic atrophy in our *Opa1v1* or *Opa1v1*Δ*4* animals, we assessed their visual capabilities by virtual drum tests and evaluated the thickness of the retina and examined the retinal fundus, alongside examining the primary blood vessels within the retina. Our findings revealed no signs of optic atrophy in either of the mouse lines (fig. S2, E to I). Together, *Opa1v1* or *Opa1v1*Δ*4* mice do not exhibit the mitochondrial dysfunction typically associated with *OPA1* mutations observed in human cases, indicating a lack of the characteristic mitochondrial disease manifestations.

### Opa1 processing is not essential under metabolic stress conditions

**Fig. 4.**
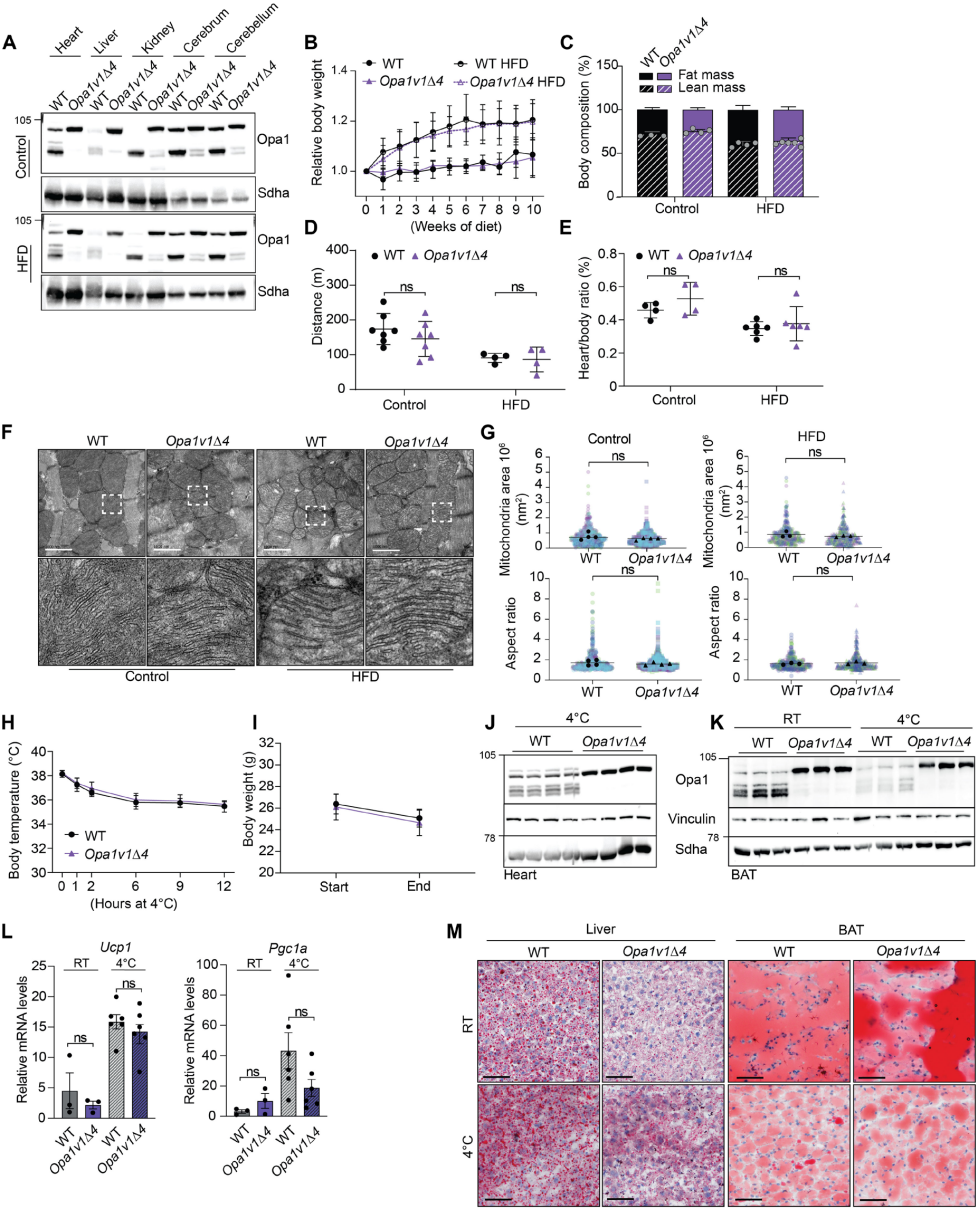
Opa1 processing is not essential upon HFD feeding or cold exposure of mice. (**A**) Feeding of 54-week-old male mice for 10 weeks with a high-fat diet (HFD) did not induce Opa1 processing in various tissues. (**B**) HFD causes similar body weight gain in WT and *Opa1v1*Δ*4* mice (WT, *n* = 6; *Opa1v1*Δ*4*, *n* = 6; WT HFD, *n* = 4; and *Opa1v1*Δ*4* HFD, *n* = 5). (**C**) HFD increased fat mass similarly in WT and *Opa1v1*Δ*4* animals (male mice, *n* = 3 to 4 in the control diet and *n* = 4 to 6 in HFD). (**D**) WT and *Opa1v1*Δ*4* mice performed similarly in treadmill analysis (male mice, *n* = 7 in the control diet and *n* = 4 in HFD). (**E**) Heart/body weight ratio was similar in WT and *Opa1v1*Δ*4* mice on both diets (standard diet, *n* = 4; and HFD, *n* = 6). (**F** and **G**) HFD did not affect mitochondrial ultrastructure in the heart as shown by TEM analysis: Four mice were analyzed on control diet (*n* = 529 WT and *n* = 658 *Opa1v1*Δ*4* mitochondria); four WT mice on HFD (*n* = 458 mitochondria) and three *Opa1v1*Δ*4* mice on HFD (*n* = 408 mitochondria). Scale bars, 1 μm. (**H** and **I**). Rectal body temperature during 12 hours of cold exposure (4°C) for 12-week-old male mice and body weight before and after 12 hours at 4°C (WT, *n* = 7; and *Opa1v1*Δ*4*, *n* = 5). (**J** and **K**) Cold exposure did not increase Opa1 processing in the heart or BAT (*n* = 3 to 4). (**L**) Uncoupling protein 1 (*Ucp1*) and *Pgc1α* mRNA levels were similar in BAT of WT and *Opa1v1*Δ*4* mice (*n* = 3 to 6). (**M**) Oil Red O staining from the liver and BAT. Cold exposure decreased the fat deposits in BAT similarly in WT and *Opa1v1*Δ*4* mice (*n* = 3). Scale bars, 50 μm. RT, room temperature.

*Oma1^−/−^* mice show increased diet-induced obesity and altered thermogenesis during cold stress (*39*). To investigate the effect of impaired Oma1-mediated Opa1 processing under metabolic stress, we treated 1-year-old WT and *Opa1v1*Δ*4* mice with a high-fat diet (HFD) for 10 weeks. HFD feeding did not induce Opa1 processing in tissues of WT or *Opa1v1*Δ*4* animals (Fig. 4A), which showed a similar increase in body weight (Fig. 4B). Analysis of the body composition of the mice showed no difference in the fat mass after HFD (Fig. 4C), and also the running performance was not altered between the mouse strains (Fig. 4D). HFD induced the expected metabolic shift toward fatty acid utilization in both WT and *Opa1v1*Δ*4* mice (fig. S2J), demonstrating that Opa1 processing is not essential for increased β-oxidation upon HFD feeding. Similarly, animal movement, estimated heat production, or food consumption was consistent between the genotypes (fig. S2, K to M). HFD can lead to hypertrophic cardiomyopathy and mitochondrial dysfunction. After 10 weeks of HFD, heart weight was unaffected and TEM analysis showed no apparent differences in mitochondrial ultrastructure between WT and *Opa1v1*Δ*4* animals (Fig. 4, E to G).

Cold exposure induces dynamic remodeling of mitochondria and Opa1 has been shown to be essential for the proper function of BAT (*12*, *39*, *49*). To investigate whether Opa1 processing is essential in thermogenic adaptation, we studied 12-week-old WT and *Opa1v1*Δ*4* mice under cold stress. We found no notable differences in body temperature or body weight between the genotypes (Fig. 4, H and I). Cold stress did not affect Opa1 levels in *Opa1v1*Δ*4* BAT, although there was a reduction in total Opa1 levels in WT BAT as observed previously (Fig. 4K) (*39*). To further analyze the thermogenic response, we measured mRNA levels of uncoupling protein 1 (*Ucp1*), which regulates mitochondrial heat production, and of the transcriptional co-activator Peroxisome proliferator-activated receptor gamma coactivator 1-alpha (*Pgc1*α) but did not observe significant differences between the mouse strains (Fig. 4L). Furthermore, cold-induced fatty acid depletion from the liver and BAT, as assessed by Oil Red O staining, was similar in WT and *Opa1v1*Δ*4* mice (Fig. 4M). We conclude from these experiments that balanced Opa1 processing is not essential under metabolic stress conditions such as HFD or cold stress.

### Opa1 processing has a protective role under severe OXPHOS stress

**Fig. 5.**
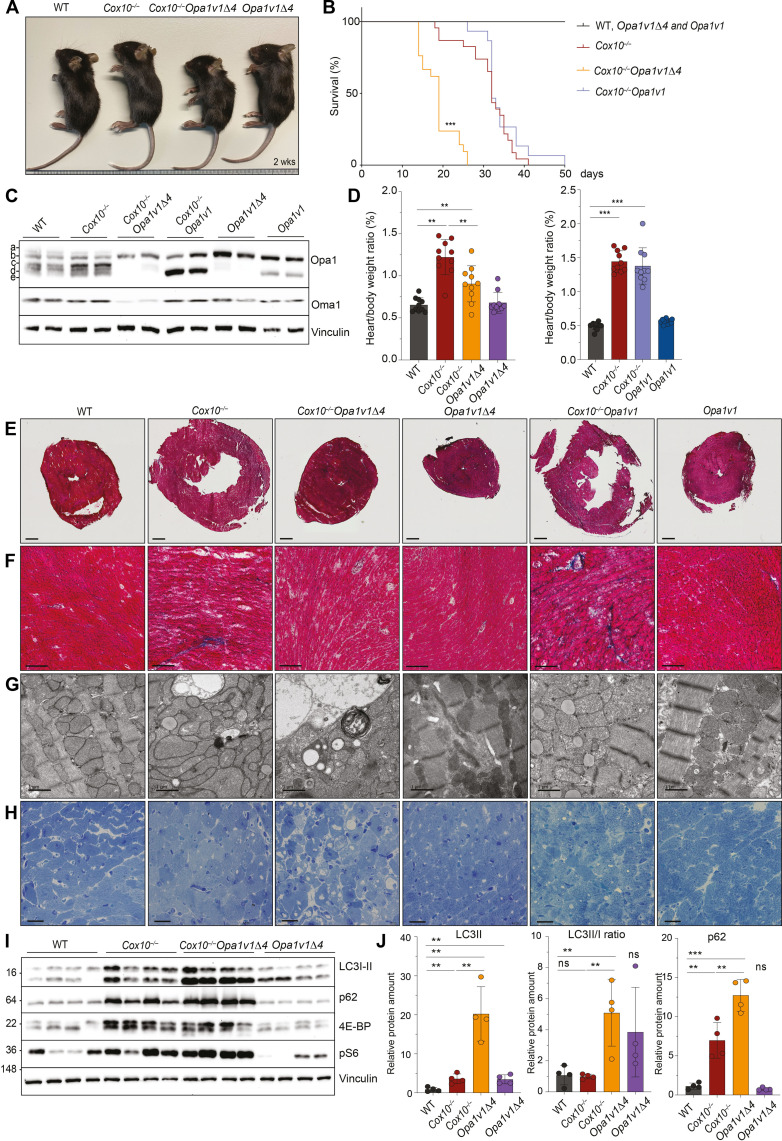
Opa1 processing is protective in mitochondrial cardiomyopathy and supports hypertrophic growth. (**A** and **B**) *Opa1v1* and *Opa1v1*Δ*4* mice were crossed with cardiac and skeletal muscle–specific knockout mice of *Cox10*. Loss of Opa1 processing markedly reduced the life span of *Cox10^−/−^* mice, whereas V1 preserves the life span of *Cox10^−/−^* mice. (**C**) Increased Opa1 processing and s-Opa1 levels in *Cox10^−/−^* and *Cox10^−/−^Opa1v1* hearts indicating Oma1 activation. Opa1 processing is abolished in *Cox10^−/−^Opa1v1*Δ*4* hearts. (**D**) *Cox10^−/−^* and *Cox10^−/−^Opa1v1* mice show an increased heart/body weight ratio, which is reduced in *Cox10^−/−^Opa1v1*Δ*4* mice*.* Heart/body weight ratios were determined in age-matched mice and are from 2-week-old (*Cox10^−/−^Opa1v1*Δ*4*) and from 3.5-week-old *Cox10^−/−^Opa1v1* mice (*n* = 10; 5 females and 5 males). (**E**) Cross-sectional images from Masson’s trichrome stained hearts showing hypertrophic growth of *Cox10^−/−^* and *Cox10^−/−^Opa1v1* hearts. Scale bars, 1 mm. (**F**) Close-up images (10×) of Masson’s trichrome-stained hearts. (**G**) Representative TEM and (**H**) toluidine blue images from hearts, showing the accumulation of distorted mitochondria in *Cox10^−/−^*, *Cox10^−/−^Opa1v1*, and *Cox10^−/−^Opa1v1*Δ*4* heart section and additionally accumulating vacuolar structures in *Cox10^−/−^Opa1v1*Δ*4* heart sections. (G) Scale bars, 1 μm. (H) Scale bars, 20 μm. (**I** and **J**) Lipidated LC3II form and p62 accumulates in *Cox10^−/−^* and, at an even higher level, in *Cox10^−/−^Opa1v1*Δ*4* hearts.

Oma1-mediated DAP3 binding cell death enhancer 1 (Dele1) processing elicits the mitochondrial integrated stress response (ISR^mt^) (*43*, *44*), which protects OXPHOS-deficient hearts against ferroptosis (*50*). As OXPHOS deficiency also induces Opa1 processing by Oma1 in vivo, we investigated the role of Opa1 processing in cardiomyopathy associated with OXPHOS dysfunction. We used cardiac and skeletal muscle–specific cytochrome c oxidase assembly factor heme A:farnesyltransferase (*Cox10^−/−^*)–deficient mice as a model for mitochondrial cardiomyopathy. *Cox10* encodes a heme A–farnesyltransferase, which is required for the assembly of the cytochrome c oxidase complex (*51*). Accordingly, loss of Cox10 in the heart leads to OXPHOS deficiency and early-onset dilated cardiomyopathy (*50*). Breeding of *Cox10^−/−^* mice with *Opa1v1* and *Opa1v1*Δ*4* mice revealed marked differences in the life span of the mice: Whereas the life span of *Cox10^−/−^Opa1v1* mice was identical to that of *Cox10^−/−^* animals (median, 32 days), *Cox10^−/−^Opa1v1*Δ*4* mice had a strongly reduced life span (median, 19 days) (Fig. 5, A and B). *Cox10^−/−^Opa1v1*Δ*4* mice developed normally and were indistinguishable from their littermates until the age of ~2 weeks when pups became less active, had a hunched posture, and died suddenly within 24 hours. As previously reported (*50*), loss of Cox10 in cardiomyocytes induced Opa1 processing by Oma1 (Fig. 5C). Processing of variant 7 generates Opa1 form c, whereas Oma1-mediated cleavage of V1 leads to the formation of Opa1 form e. Accordingly, Opa1v1 mice accumulate only form b (corresponding to the non-cleaved form of V1) and form e generated by Oma1 (Fig. 5C). V1 processing and the level of Opa1 form e were increased in *Cox10^−/−^Opa1v1* hearts*.* In contrast, V1Δ4 stably accumulated even in OXPHOS deficiency, although formation of minute amounts of s-Opa1 cannot be excluded (Fig. 5C). Opa1 protein levels were modestly decreased, suggesting increased degradation of V1Δ4 during OXPHOS stress. Similarly, Oma1 accumulated at lower levels in *Cox10*^−/−^ hearts, likely reflecting autocatalytic turnover of Oma1 under OXPHOS stress (Fig. 5C) (*21*).

Loss of Cox10 in the heart results in cardiac hypertrophy (Fig. 5, D to F) (*50*). The heart/body weight ratio of *Cox10^−/−^Opa1v1* mice was indistinguishable from that of *Cox10^−/−^* animals (Fig. 5D). However, the hypertrophic growth of *Cox10^−/−^Opa1v1*Δ*4* hearts was significantly reduced when compared to those in *Cox10^−/−^* animals (Fig. 5, D to F). These observations indicate that V1 is functional and that Oma1-mediated Opa1 processing is required for cardiac hypertrophy induced by OXPHOS deficiency. Notably, impaired Opa1 processing did not affect Oma1- and Dele1-dependent ISR^mt^, which protects OXPHOS-deficient cardiomyocytes from ferroptosis (*50*). We observed similar ISR^mt^ activation, similar levels of Gpx4, and a slight accumulation of lipid peroxides in *Cox10^−/−^* and *Cox10^−/−^Opa1v1*Δ*4* hearts (fig. S3, A to E). Thus, the requirement of Oma1-mediated Opa1 processing for cardiac hypertrophy is independent of mitochondrial stress signaling.

### Loss of Opa1 processing in *Cox10^−/−^* mice deregulates autophagy

Although cardiac hypertrophy was reduced, analysis of *Cox10^−/−^Opa1v1*Δ*4* hearts by fluorescence and electron microscopy revealed severely disrupted cardiac tissue (Fig. 5, F and H, and fig. S3F) and enlarged mitochondria (Fig. 5G). We observed large vacuolar, possibly autolysosomal structures, reminiscent of our earlier observations in *Cox10^−/−^Oma1^−/−^* hearts (Fig. 5, G and H) (*50*). OXPHOS deficiencies were previously found to impair lysosomal functions via diverse mechanisms (*52*–*54*), which may interfere with autophagy. Consistently, lipidated microtubule-associated protein 1A/1B light chain 3 (LC3II), which accumulates in lysosome-targeted membranes and drives the autophagosome maturation, was significantly increased in *Cox10^−/−^Opa1v1*Δ*4* hearts (Fig. 5, I and J). We also observed accumulation of the autophagy receptor sequestosome 1 (p62) in heart lysates by immunoblotting (Fig. 5, I and J) and by immunohistochemical staining of *Cox10*^−/−^ and *Cox10^−/−^Opa1V1*Δ*4* hearts (fig. S3, G and H), pointing to an overall increased autophagic flux or stalled autophagy, which appeared further aggravated in *Cox10^−/−^Opa1v1*Δ*4* hearts compared to that in *Cox10*^−/−^ hearts. LC3 lipidation was similar in *Cox10*^−/−^ and *Cox10^−/−^Opa1V1* hearts, highlighting the role of the Opa1 processing (fig. S3I). In agreement with autophagy inhibition, phosphorylation of mTORC1 substrates, the ribosomal protein S6 and the eukaryotic initiation factor 4E binding protein 1 (4E-BP1), were increased in hearts lacking Cox10 (Fig. 5I). To assess how impaired Opa1 processing affects the autophagic flux, we isolated MEFs from WT, *Cox10^−/−^*, *Opa1v1*Δ*4*, and *Cox10*^−/*−*^*Opa1v1*Δ*4* mice. Similar to cardiomyocytes in vivo, loss of Cox10 induces Opa1 processing in MEFs, which was inhibited in cells harboring V1Δ4 (fig. S3J). Inhibition of autophagosome fusion with chloroquinone caused accumulation of LC3 in WT and *Cox10*^−/−^ MEFs as expected (fig. S3K). In contrast, LC3 lipidation was inhibited in *Opa1v1*Δ*4* and *Cox10^−/−^Opa1v1*Δ*4* MEFs (fig. S3K). These results indicate that suppression of Opa1 processing in Cox10-deficient cells expressing Opa1v1Δ4 impairs autophagy in vivo and in vitro, although inhibition of this process occurs apparently at an earlier stage in vitro. Thus, autophagic deficiencies may limit the removal of dysfunctional mitochondria and aggravate consequences of cardiac OXPHOS deficiency in *Cox10^−/−^Opa1v1*Δ*4* mice.

### *Cox10^−/−^* and *Cox10^−/−^Opa1v1*Δ*4* hearts show hypertrophic gene expression

**Fig. 6.**
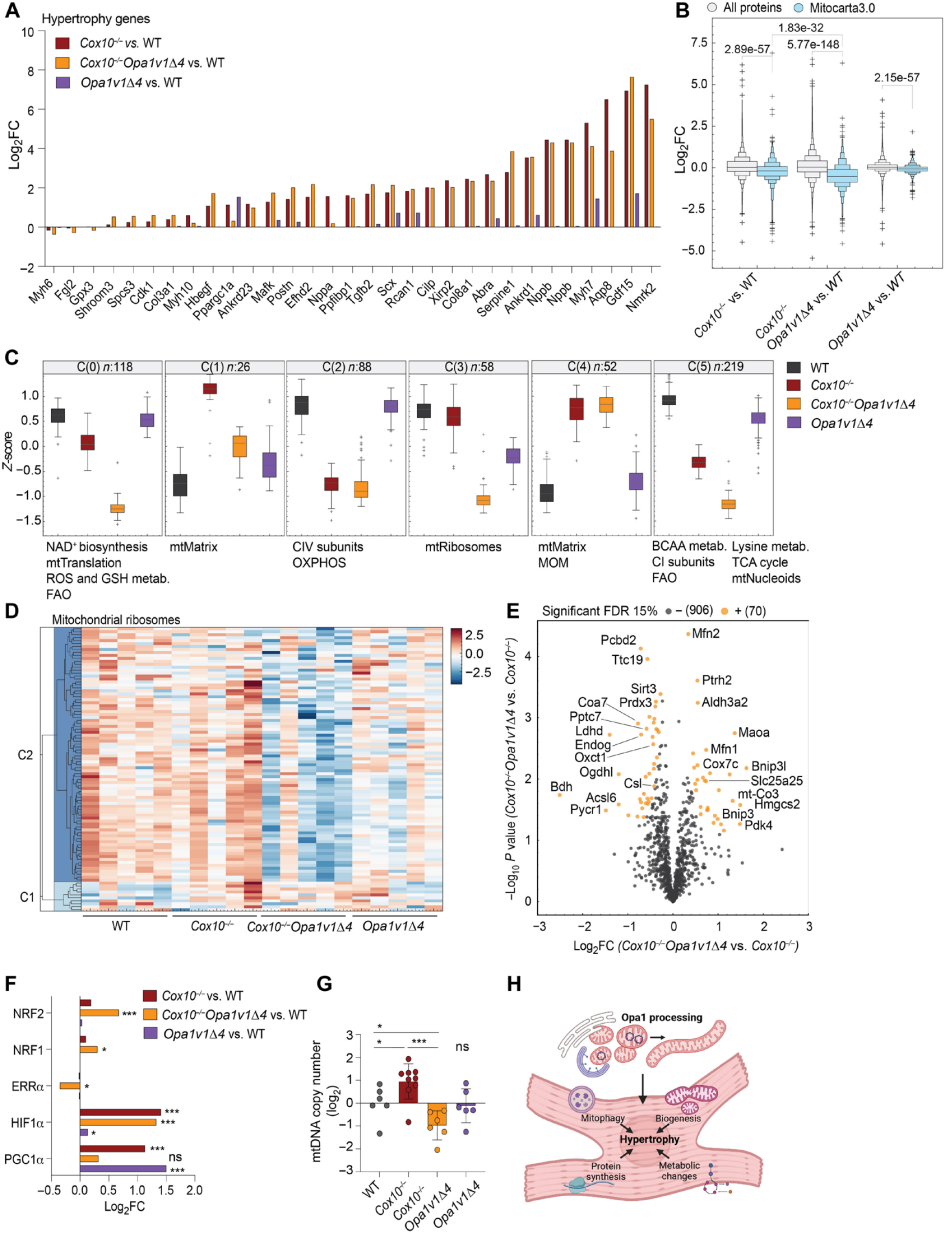
Impaired mitochondrial biogenesis in *Cox10^−/−^Opa1v1*Δ*4* hearts. (**A**) RNA-seq analysis of 2-week-old WT, *Cox10^−/−^*, *Cox10^−/−^Opa1v1*Δ*4*, and *Opa1v1*Δ*4* mice revealed increased expression of hypertrophic genes both in *Cox10^−/−^* and *Cox10^−/−^Opa1v1*Δ*4* hearts. Hypertrophic genes were annotated according to (*55*). (**B**) Analysis of heart proteomes showed significant down-regulation of mitochondrial proteins (MitoCarta3.0) in *Cox10^−/−^* and *Opa1v1*Δ*4* hearts that was aggravated in *Cox10^−/−^Opa1v1*Δ*4* hearts (*n* = 5). A Mann-Whitney *U* test was used to compare the log_2_ fold change (log_2_FC) distributions (**C**) *K*-means clustering result of the *z*-score–transformed LFQ intensities (six clusters). Significantly enriched Gene Ontology (GO)/MitoCarta3.0 pathways (FDR < 0.02, Fisher exact test) are listed below each cluster. The title indicates the cluster index and the number of proteins. ROS, reactive oxygen species; GSH, glutathione; FAO, fatty acid oxidation; BCAA, branched-chain amino acids; CI, complex I; CIV, complex IV; MOM, mitochondrial outer membrane; TCA, tricarboxylic acid cycle. (**D**) Heatmap of *z*-scores of transformed LFQ intensities of proteins annotated to the GO term “mitochondrial ribosome.” (**E**) Volcano blot of mitochondrial proteins after normalization to mitochondrial mass in *Cox10^−/−^Opa1v1*Δ*4* versus *Cox10^−/−^* hearts (Mitocarta3.0). (**F**) Relative expression (fold change) of selected transcription factors in mouse heart RNA-seq dataset (A) shown as log_2_FC. (**G**) Relative mtDNA levels in the heart of 2-week-old mice determined by qPCR for *cytB*. (**H**) Schematic presentation of the role of Opa1 processing in cardiac hypertrophy induced by OXPHOS dysfunction. Scheme was created with BioRender.com.

To better understand the absence of hypertrophic growth of *Cox10^−/−^Opa1V1*Δ*4* hearts, we performed an RNA sequencing (RNA-seq) analysis and examined the expression of genes characteristic of cardiac hypertrophy (*55*). Notably, loss of Opa1 processing in *Cox10*^−/−^ hearts did not significantly affect hypertrophic gene expression (Fig. 6A). Hypertrophic signature genes were up-regulated relative to WT in the hearts of both *Cox10^−/−^* and *Cox10^−/−^Opa1v1*Δ*4* mice, although we did not observe cardiac hypertrophy in the latter mouse line (Fig. 5, D and E). Autophagy-related genes were similarly affected in *Cox10^−/−^* and *Cox10^−/−^Opa1v1*Δ*4* mice (fig. S3L). Moreover, an ingenuity pathway analysis identified the up-regulation of similar cellular pathways and upstream regulatory genes, such as eukaryotic translation initiation factor 2 α kinase 3 (*Eif2ak3* encoding Dele1) (fig. S4, A and B). These data further corroborate that the ISR^mt^ does not depend on Opa1 processing (fig. S3, A and B). Comparison of gene expression profiles of *Cox10^−/−^Opa1v1*Δ*4* and *Cox10^−/−^* hearts identified target genes of clustered mitochondria homolog (Cluh) as being most significantly down-regulated upon inhibition of Opa1 processing (fig. S4C). Cluh is an RNA-binding protein that stabilizes the mRNA of nuclear-encoded mitochondrial proteins and controls their translation (*56*). Loss of *Cluh* leads to down-regulation of catabolic pathways required under starvation and inhibition of OXPHOS (*57*), while a protective effect of Cluh overexpression has recently been observed in a model of cardiac hypertrophy (*58*).

### *Cox10^−/−^Opa1v1*Δ*4* hearts fail to maintain mitochondria

These observations prompted us to analyze the heart proteome of WT, *Cox10*^−/−^, *Opa1v1*Δ*4*, and *Cox10^−/−^Opa1v1*Δ*4* mice using a data-independent (DIA) mass spectrometry–based proteomics approach. In total, we were able to quantify 10,051 protein groups including 991 MitoCarta3.0 positive protein groups and statistical analysis [one-way analysis of variance (ANOVA), false discovery rate (FDR) < 0.05] revealed 3500 significantly changed proteins, out of which 562 were mitochondrial (table S2). The analysis of log_2_ fold change distributions of all proteins (relative to WT) against mitochondrial protein groups revealed overall decreased levels of mitochondrial proteins in *Cox10^−/−^* and *Cox10^−/−^Opa1v1*Δ*4* hearts compared to those in WT hearts [according to MitoCarta3.0; (*59*)], which was even more pronounced in the absence of Opa1 processing (Fig. 6B).

We then performed a *K*-means clustering based on the *z*-score transformed log_2_ label-free quantification (LFQ) intensities of the significantly regulated mitochondrial (MitoCarta3.0) protein groups (*N*_Cluster_ = 6) (Fig. 6C). This analysis revealed three distinct clusters (C0, C3, and C5) with a *Cox10^−/−^Opa1v1*Δ*4*-dependent regulation. Gene Ontology/MitoCarta3.0 pathway enrichment revealed that several mitochondria-related metabolic pathways, such as nicotinamide adenine dinucleotide (oxidized form) (NAD^+^) metabolism, branched-chain amino acid metabolism, fatty acid oxidation, mitochondrial fatty acid synthesis, or tricarboxylic acid cycle, were found at decreased levels in *Cox10^−/−^Opa1v1*Δ*4* hearts compared to those in *Cox10*^−/−^ hearts (cluster 0, *n* = 118, Fig. 6C). In addition, a down-regulation of mitochondrial ribosomes was observed (cluster 3, *n* = 58) (Fig. 6, C and D). Impaired Opa1 processing did affect neither the *Cox10* gene deletion–mediated loss of subunits of complex IV nor other OXPHOS complexes in cardiac mitochondria (cluster 2, *n* = 88, Fig. 6C and fig. S4D), which show impaired assembly and activity of cytochrome c oxidase (COX) complexes (fig. S4, E and F). Similarly, we observed the accumulation of cytoplasmic ribosomes both in *Cox10*^−/−^ and *Cox10^−/−^Opa1v1*Δ*4* hearts, likely reflecting a compensatory response to OXPHOS deficiency (fig. S4G). We conclude from these studies that *Cox10^−/−^Opa1v1*Δ*4* hearts fail to maintain mitochondrial metabolic functions. Because cardiac hypertrophy is caused by an increase in myocyte size, which depends on mitochondrial proliferation, the reduced mitochondrial mass in *Cox10^−/−^Opa1v1*Δ*4* hearts may limit hypertrophic cell growth.

To identify proteins whose steady-state levels specifically changed in response to impaired Opa1 processing, we normalized our proteomic data to mitochondrial mass, which was overall reduced in *Cox10^−/−^Opa1v1*Δ*4* hearts (Fig. 6B). This analysis showed that the mitophagy receptors Bcl2-interacting protein 3 (Bnip3) and Bnip3-like (Bnip3l and Nix) as well as mitofusins Mfn1 and Mfn2 accumulated in *Cox10^−/−^Opa1v1*Δ*4* heart when compared to those in *Cox10^−/−^* heart (two-sided *t* test, permutations 500; FDR < 15%). The accumulation of mitophagy receptors and decreased levels of the mitochondrial protein phosphatase targeting COQ7 (Pptc7), which has been shown to regulate mitophagy receptor stability (*60*, *61*), further supports our hypothesis of deregulated mitophagy in *Cox10^−/−^Opa1v1*Δ*4* heart. However, our results do not exclude that alterations in the biogenesis of mitochondria contribute to the decreased mitochondrial mass in the heart of these mice. The mRNA levels of the key transcriptional coactivator Pgc1α were similar in WT and *Cox10^−/−^Opa1v1*Δ*4* hearts but increased in *Cox10*^−/−^ hearts (Fig. 6F). On the other hand, *Cox10^−/−^Opa1v1*Δ*4* showed increased levels of the nuclear respiratory factors Nrf1 and Nrf2 and down-regulation of estrogen related receptor, alpha (ERRα), indicating dysregulation of mitochondrial biogenesis (Fig. 6F). Consistently, loss of Opa1 processing strongly reduced mtDNA levels in *Cox10^−/−^Opa1v1*Δ*4* mitochondria, while *Cox10*^−/−^ hearts showed increased mtDNA levels (Fig. 6G). Together, our proteome and transcriptome analysis of *Cox10^−/−^* and *Cox10^−/−^Opa1v1*Δ*4* hearts suggests that disbalanced mitochondrial biogenesis and mitophagy limit cardiac hypertrophy (Fig. 6H), which is primarily a compensatory reaction to physiological stress, but almost inevitably leads to decompensation if stress persists, resulting eventually in cardiac failure.

## DISCUSSION

Increased Opa1 processing promotes mitochondrial fragmentation under various stress conditions. The establishment of knock-in mouse models expressing exclusively a cleavable or non-cleavable Opa1 variant 1 (V1, V1Δ4) allowed the analysis of the physiological role of different Opa1 splice variants and of stress-induced Opa1 processing without confounding effects of the loss of pleiotropic processing peptidases. Our analysis shows that the expression of different Opa1 isoforms and Opa1 cleavage by Oma1 and Yme1l are dispensable for normal mouse development and health. In contrast, Oma1-mediated Opa1 processing limits mitochondrial cardiomyopathy in OXPHOS-deficient hearts, highlighting the importance of Opa1 cleavage and of s-Opa1 in mitochondrial heart disease.

Although four different splice variants of Opa1 are expressed in mice, *Opa1v1* mice expressing only V1 are healthy and develop normally. *Opa1v1* mice show normal metabolic parameters and visual functions compared to WT mice. While isoform-specific Opa1 functions may exist (*31*, *45*), they do not appear to contribute to the functional deficits observed in the absence of Opa1 in vivo. Similarly, because V1 lacks Yme1l processing sites, Yme1l-mediated processing is not required under normal physiological conditions or under metabolic stress of HFD feeding or cold stress. It is conceivable that Yme1l regulates Opa1 cleavage or overall Opa1 protein levels only in some cells or under specific stress conditions, such as in starvation and hypoxia, when Yme1l-mediated proteolysis limits the accumulation of Opa1 (*26*).

Increased cleavage of Opa1 by the stress-activated peptidase Oma1 drives mitochondrial fragmentation and contributes to mitochondrial quality control (*62*). Unexpectedly, however, abrogation of Oma1-mediated Opa1 cleavage by deletion of four amino acids at S1 did not disrupt normal mouse development. We observed a strong impairment of Opa1 processing in all tissues in *Opa1v1*Δ*4* mice, which was completely blocked in tissues, such as the heart, liver, and BAT. The absence of any phenotypes reminiscent of patients with ADOA with *OPA1* mutations in *Opa1v1*Δ*4* mice suggests that deficiencies in OPA1 processing and the absence of s-Opa1 do not contribute to the pathology of the disease. In addition, *Opa1v1*Δ*4* mice did not show increased diet-induced obesity or altered thermogenesis during cold stress, as has been observed in *Oma1*^−/−^ mice (*39*). Therefore, these phenotypes appear to be unrelated to impaired Opa1 processing and may reflect defects in mitochondrial stress signaling or other metabolic functions in the absence of Oma1 (*40*, *43*, *44*, *50*).

The lack of gross phenotypes in *Opa1v1*Δ*4* mice contrasts with the severe consequences of mitochondrial fusion and fission defects (*63*). Our results are consistent with in vitro studies, showing that l-Opa1 is sufficient to promote mitochondrial fusion (*20*, *27*, *28*), although the presence of s-Opa1 further stimulates membrane fusion activity (*28*, *29*). This is remarkable considering the boost of mitochondrial dynamics during the development of some tissues such as the heart (*64*). Accumulation of l-Opa1 in the heart of *Opa1v1*Δ*4* mice preserved OXPHOS activity and mitochondrial ultrastructure, consistent with observations in mice lacking both Yme1l and Oma1 in cardiomyocytes (*35*). We only observed mild changes in cristae density in cardiac mitochondria using TEM. This is in agreement with a recent cryo–electron tomographic analysis of mitochondrial cristae shapes in cells accumulating only l-Opa1 (*65*). It should be noted, however, that our in vivo analysis did not distinguish between lamellar and tubular cristae, the ratio of which has been shown to be affected by Opa1 and Opa1 cleavage (*65*, *66*).

While dispensable for normal mouse development, inhibition of Opa1 processing aggravated the mitochondrial cardiomyopathy caused by the loss of Cox10 in cardiomyocytes. We observed a shortened life span of Cox10-deficient mice in the presence of V1Δ4 but not V1, demonstrating that Opa1 processing and s-Opa1 formation exert a protective function in the OXPHOS-deficient heart. Notably, inhibition of Opa1 processing does not interfere with Oma1-mediated Dele1 processing and the induction of the ISR^mt^, showing that Oma1 confers protection to the heart by the concerted regulation of both mitochondrial stress signaling and mitochondrial dynamics. The deleterious effect of impaired Opa1 processing in the OXPHOS-deficient heart may be explained by the limited fusion capacity of l-Opa1 in the absence of s-Opa1, although fusion (and fission) events are very rare in adult cardiac myocytes (*63*). Furthermore, we observed deregulated autophagic processes in Cox10-deficient hearts when Opa1 processing is impaired. Consistent with stalled mitophagy, we observed accumulation of lipidated LC3, of the autophagy receptor p62, and of the mitophagy receptors Bnip3l and Bnip3, as well as perinuclear clustering of mitochondria and prolonged interactions of mitochondria with the ER. Balanced Opa1 processing ensures mitochondrial dynamics. It is conceivable that the accumulation of l-Opa1, which promotes fusion, might limit mitophagy. However, we cannot exclude other mechanisms, such as prolonged ER-mitochondria contact sites. Damaged mitochondria may, therefore, exacerbate mitochondrial cardiomyopathy, but additional defects that are associated with the loss of Opa1 processing appear to contribute.

It should be noted that defects caused by the impaired Opa1 processing in Cox10-deficient hearts differ from previous mouse models lacking mitochondrial dynamic components in the heart, which may be explained by the detrimental effect of an OXPHOS deficiency on lysosomal functions (*52*–*54*). Inhibition of fission by deletion of dynamin-related protein 1 (*Drp1*) increases mitophagy and caused a loss of mitochondria in the heart (*67*). On the other hand, cardiac *Mfn2* ablation interrupts the autophagic removal of mitochondria from cardiomyocytes and leads to the accumulation of mitochondria without increasing mitophagy (*63*, *67*). These studies revealed that mitochondrial dynamics factors orchestrate mitochondrial biogenesis and mitophagy, the balance of which is critical for cardiac function (*68*). Consistently, our proteomic analysis showed decreased rather than increased mitochondrial protein levels in hearts lacking Cox10 and Opa1 cleavage despite impaired mitophagy, suggesting deficiencies in mitochondrial biogenesis. Cardiac hypertrophy is associated with increased myocyte growth, which depends on various mitochondrial functions (*69*–*71*). Reduced mitochondrial biogenesis could, therefore, explain why the loss of Opa1 processing impairs the hypertrophic growth of Cox10-deficient hearts, despite the induction of the hypertrophic gene expression programme. How the cleavage of Opa1 and the accumulation of s-Opa1 affect mitochondrial biogenesis remains to be elucidated. Regardless, our results reveal an intriguing regulatory circuit linking Opa1 processing to cardiac hypertrophy and highlight the role of Oma1 as a stress-protective protease in heart disease (*35*, *50*, *72*).

## MATERIALS AND METHODS

### Animal studies

All animal work was approved by local authorities (Landesamt für Natur, Umwelt und Verbraucherschutz Nordrhein-Westfalen, Germany), and animal procedures were carried out in accordance with European, national, and institutional guidelines and according to good practice of animal handling. Mice were maintained at the specific pathogen–free animal facility of the Max Planck Institute for Biology of Ageing with 12-hour light cycle and regular chow diet. *Opa1v1* and *Opa1v1*Δ*4* mice were created with the CRISPR-Cas9 system by electroporation of mouse zygotes with a NEPA21 Electroporator (CUY501P1-1.5 electrode) and the IDT Alt-R CRISPR-Cas9 System (Integrated DNA Technologies Inc.) with gRNAs and repair oligonucleotides listed in table S1. The Ckmm-Cre-*Cox10*^fl/fl^ mice expressing cardiac and skeletal muscle–specific knockout of *Cox10* in the C57BL/6N background were previously published by us (*50*). Groups included male and female animals. Samples for protein and RNA extraction were taken after cervical dislocation and snap-frozen in liquid nitrogen. We used age-matched mice for protein, metabolome, and RNA analysis as well as histological analysis. For the cold-stress study, we used 12-week-old mice and kept them in 4°C for 12 hours with a rectal body temperate monitoring after 1, 2, 6, 9, and 12 hours. HFD (ssniff D12492) was applied to 54-week-old mice with a duration of 10 weeks. The diet consisted of 60 kJ% of fat (lard and soybean oil), 20 kJ% of proteins, and 20 kJ% of carbohydrates. HFD-fed and control animals were analyzed in a Phenomaster for respiratory exchange ratio (RER), food consumption, movement, and estimated heat production for a duration of 48 hours. At the German Mouse Clinic, mice were maintained in individually ventilated cages (IVC) with water and standard mouse chow according to the directive 2010/63/EU, German laws, and German Mouse Clinic (GMC) housing conditions (www.mouseclinic.de). All tests were approved by the responsible authority of the district government of Upper Bavaria. A cohort of 21 Opa1v1 mice (12 males/9 females) with corresponding controls (9 males/13 females) and 29 Opa1v1delta4 mice (15 males/14 females) with corresponding controls (14 males/14 females) entered the phenotypic pipeline at 68 weeks of age. Animal numbers may vary depending on the test performed, as indicated in the respective figure or table. The phenotypic tests were part of the GMC screening pipeline and performed according to standardized protocols as described before (*73*–*75*).

### Histology

Animals were euthanized, tissues were rinsed in ice-cold phosphate-buffered saline (PBS) and fixed in 4% paraformaldehyde at 4°C for 72 hours. Paraffin-embedded tissues were cut into 5-μm sections deparaffinized in xylol, rehydrated and stained with hematoxylin and eosin or with Masson’s trichrome, and imaged using 10× dry objective at Eclipse Ci histology microscope (Nikon Eclipse).

Hearts were snap-frozen in isopentane chilled with liquid nitrogen, cut into 10-μm sections with a cryostat, and stained for COX and/or succinate dehydrogenase (SDH) activity. Heart sections were incubated in standard COX solution for 12 min at room temperature (RT) and/or incubated in SDH solution for 5 min at 37°C. Oil Red O staining was performed for frozen sections after fixing the sections with a formalin-Ca^2+^ solution and stained in Oil Red O for 10 min. Nuclei were stained with Mayer’s hematoxylin, and sections were mounted with aqueous mounting medium. For immunofluorescence staining, frozen sections were cut into 8-μm sections, blocked with 5% horse serum for 1 hour, and incubated overnight at 4°C in 2% bovine serum albumin (BSA) with selected antibodies: malondialdehyde (Abcam, ab243066) and p62 (Abnova, H00008878).

### SDS-PAGE, BN, and immunoblot analysis

Mouse heart tissue was homogenized with Precellys 24 tissue homogenizer (Bertin Instruments) two times for 20 s at 6000 rpm in protein lysis buffer [50 mM tris-HCl (pH 7.5), 150 mM NaCl, 5 mM MgCl_2_, 1 mM dithiothreitol, 10% glycerol, 2% SDS, and 1% Triton X-100] containing protease inhibitor cocktail (Roche) and phosphatase inhibitor cocktail (PhosSTOP, Roche). Protein concentration was determined with bicinchoninic acid (BCA) protein assay (Pierce). For protein extraction from MEFs, cells were washed with cold PBS and resuspended in ice-cold radioimmunoprecipitation assay buffer [50 mM tris-HCl (pH 7.4), 150 mM NaCl, 1% Triton X-100, 0.1% SDS, 0.05% sodium deoxycholate, and 1 mM EDTA] containing protease inhibitor cocktail (Roche) and phosphatase inhibitor cocktail (PhosSTOP, Roche). Samples were boiled before SDS-PAGE in 95°C for 5 min except in a case of OXPHOS subunit analysis. Total proteins from tissue or cells (25 to 50 μg) were separated using SDS-PAGE, followed by transfer to nitrocellulose membranes and immunoblotting with the following antibodies: Opa1 (BD Biosciences, 612607), Oma1 (Santa Cruz Biotechnology, sc-515788) SdhA (Abcam, ab14715), vinculin [Cell Signaling Technology (CST), no. 4650], tubulin (Sigma-Aldrich, T6074), p62 (Abnova, H00008878), P-4E-BP (CST, 2855), P-S6 (CST, no. 2211 L), Atf4 (CST, no. 11815), Chop/Ddit3 (CST, no. 2895), P-eIF2alpha (Abcam, ab32157), Mthfd2 (Proteintech, 12270-1-AP), Gpx4 (Abcam, ab105066), LC3I-II (CST, no. 2775S), and Atg5 (CST, no. 2630). Western blot images were acquired with Intas ChemoStar ECL Imager HR 6.0 and ChemoStar TS software (Intas).

For BN electrophoresis analysis, we isolated mitochondria from heart tissue with incubating in 2.5% trypsin for 10 min and then homogenized in homogenization buffer [225 mM sucrose, 1 mM EGTA, 0.2% BSA, 20 mM tris-HCl (pH 7.2), and protease inhibitor cocktail (Roche)] with 10 strokes using a glass Teflon homogenizer at 1000 rpm on ice. The homogenates were centrifuged at 1000*g* for 10 min at 4°C, and the supernatant was collected. Homogenization was repeated twice. Mitochondrial fraction was isolated with centrifugation at 8000*g* for 10 min at 4°C. Protein concentration was determined using a Bradford assay. Mitochondria were solubilized with digitonin (6 g/g), and proteins were separated by native PAGE using 3 to 12% gradient gels (Invitrogen) and incubated with COX activity buffer [0.05 M phosphate buffer (pH 7.4) with diaminobenzidine (0.5 mg/ml), catalase (20 μg/ml), cytochrome c (1 mg/ml), and 220 mM sucrose] and afterward with complex I activity reagents [2 mM tris-HCl (pH 7.4) with reduced form of NAD^+^ (0.1 mg/ml) and Nitro blue tetrazolium chloride (2.5 mg/ml)].

### Electron microscopy

One- to 2-mm piece from heart tissue was fixed in 2% formaldehyde/2% glutaraldehyde in 0.1 M cacodylic acid at least 48 hours at 4°C. Samples were then washed four times for 15 min in 0.1 M cacodylic acid and fixed with 2% osmiumtetroxid (Science Services) in 0.1 M cacodylic acid and washed again four times for 15 min in 0.1 M cacodylic acid. After changes in ethanol 50 to 100%, a mixture ethanol/propyleneoxid and 100% propyleneoxid the tissue was embedded in Epon fixative. Fixed tissue was cut in 70-nm sections on the ultramicrotome (UC6, Leica) on a grid and contrasted with 1.5% uranylacetate aqueous solution for 15 min at 37°C. Cuts were washed five times in water, incubated 4 min in lead citrate, and washed again five times in water and dried on a filter paper. Images were acquired with a transmission electron microscope (JEM 2100 Plus, JEOL), a OneView 4K camera (Gatan) with DigitalMicrograph software at 80 kV at room temperature.

WT and *Opa1v1*Δ*4* MEFs were grown on ACLAR–Fluoropolymer fil (Plano) and fixed by immersion using 2% glutaraldehyde in 0.1 M cacodylate buffer at pH 7.4. After postfixation using 1% osmiumtetroxid (Science Services) in 0.1 M cacodylic acid and pre-embedding staining with 1% uranylacetate (aqueous), tissue samples were dehydrated and embedded in Agar 100. Ultrathin sections (80 nm) were counterstained using 1% uranyl acetate (aqueous) and examined using a Talos L120C (Thermo Fisher Scientific). The area and mitochondria-ER contact sites were analyzed using Fiji.

### Protein digestion for proteomics

Heart samples were lysed in 4% SDS in 100 mM Hepes-KOH (pH 8.5) using the Precellys tissue homogenizer for mechanical disruption of the tissue (according to the manufacturer’s instructions). Protein (10 μg) was subjected for tryptic digestion. Proteins were reduced [10 mM tris(2-carboxyethyl)phosphine (TCEP)] and alkylated [20 mM chloarcetamide (CAA)] in the dark for 45 min at 45°C. Samples were subjected to SP3-based digestion protocol (*76*). Washed SP3 beads [Sera-Mag magnetic carboxylate modified particles (hydrophobic and hydrophilic) from Thermo Fisher Scientific] were mixed equally, and 3 μl of bead slurry was added to each sample. Acetonitrile was added to a final concentration of 50% and washed twice using 70% ethanol (*V* = 200 μl) on an in-house–made magnet. After an additional acetonitrile wash (*V* = 200 μl), 5 μl of digestion solution [10 mM Hepes-KOH (pH 8.5) containing 0.5 μg of trypsin (Sigma-Aldrich) and 0.5 μg of LysC (Wako)] was added to each sample and incubated overnight at 37°C. Peptides were desalted on a magnet using 2× 200 μl of acetonitrile and eluted using 5% dimethyl sulfoxide (DMSO) before 60 μl of 0.1% formic acid was added. Then, the peptides were further subjected to the StageTip technique (*77*) using SDB-RP [Styrenedivinylbenzene–Reversed Phase Sulfonate (Affinisep, France)] material.

### Liquid chromatography and mass spectrometry for heart proteomics

Instrumentation consisted of an nLC-1200 (Thermo Fisher Scientific) coupled via the IonFlex electrospray source to an Exploris 480 mass spectrometer. For peptide separation, an Aurora (50 cm, 1.7-μm C18 bead size) was used at a column temperature of 55°C and a binary buffer (A) 0.1% formic acid and (B) 0.1% formic acid in 80% acetonitrile. The gradient was applied at a flow rate of 185 nl/min as follows: The content of buffer B was linearly raised from 5% to 32% in 100 min, followed by an increase to 50% within 10 min and then to 65% within 4 min. The column was washed at 70% B for 6 min.

The mass spectrometer operated in a data-independent mode and the FAIMS (high field asymmetric waveform ion mobility spectrometry) interface was attached and operated at a compensation voltage of −45. The inner electrode temperate was set to 100°C and the outer electrode was heated to 90°C. The total carrier flow was set to 3.5 liter/min. MS1 spectra were recorded in a range of 450 to 850 mass/charge ratio (*m*/*z*) using a resolution of 30,000 at 200 *m*/*z*. The radio frequence (RF) lens was set to 45%, and the spectra were recorded in profile mode. The MS2 spectra were recorded in a range of 500 to 740 *m*/*z* using an isolation window of 7 *m*/*z* and a window overlap of 1 *m*/*z*, resulting in a total number of scans of 34. The first mass was fixed to 200 *m*/*z*, and the resolution was set to 30,000 at 200 *m*/*z*. The automatic gain control (AGC) target was set to 700%, and the maximum injection time was set to “auto.” The data were acquired in centroid mode.

### Analysis of proteomic data

DIA-NN (1.8.0) (Data independent acquisition–neuronal network) (*78*) was used to analyze data-independent raw files. The spectral library was created using the uniport reference *Mus musculus* protein proteome (one sequence per gene, UniProt, number of sequences: 21,984, UP000000589, downloaded June 2022) with the “Deep learning-based spectra and RTs prediction” turned on. Protease was set to trypsin, and a maximum of one missed cleavage was allowed. N-terminal methionine excision was set as a variable modification, and carbamidomethylation at cysteine residues was set as a fixed modification. The peptide length was set to 7 to 30 amino acids and the precursor *m*/*z* range was defined as 400 to 800 *m*/*z*. The option “Quantitative matrices” was enabled.

The FDR was set to 1%, and the mass accuracy (MS2 and MS1) as well as the scan window was set to 0 (automatic inference via DIA-NN). Match between runs was enabled. The neuronal network classifier worked in “double pass mode” and protein interference was set to “Isoform IDs.” The quantification strategy was set to “robust LC (high accuracy),” and cross-run normalization was defined as “RT-dependent.”

The “pg” (protein group) output (MaxLFQ intensities) (*79*) was further processed using Instant Clue (v.12.2) (*80*). In detail, pairwise comparisons were calculated using a two-sided *t* test using the log_2_-transformed LFQ intensities, and the FDR was controlled using a permutation-based approach [Statistical analysis of micro array (SAM); #permutations, 500; FDR < 5%; s0, 0.1]. In addition, a one-way ANOVA was used to identify significantly different abundant proteins in any group of the four genotypes (#permutations, 500; FDR < 5%; s0, 0.1). MitoCarta3.0 proteins were annotated, and a *k*-means clustering (*z*-score–transformed) was performed after filtering for ANOVA significance and MitoCarta3.0 protein groups (FDR < 0.05) using a total number of six clusters (determined by the “elbow” method) using Instant Clue (*80*). Pathway enrichment (MitoCarta Mito Pathways and Gene Ontologies) was assessed using a Fisher exact test followed by Benjamini-Hochberg FDR adjustments (FDR < 2%). Log_2_-transformed LFQ intensities were normalized using the *z*-score (unit variance) and visualized in a heatmap using Euclidean distance and the complete method for the row-wise clustering.

### Cell culture, transfection, and RNA interference

SV40-immortalized monoclonal MEFs from WT, *Opa1v1*, and *Opa1v1*Δ*4* mice were maintained in Dulbecco’s modified Eagle’s medium (DMEM)–GlutaMAX (Gibco) containing glucose (4.5 g liter^−1^) supplemented with 10% fetal bovine serum (Sigma-Aldrich). *Oma1^−/−^* and corresponding WT MEFs were previously described (*20*). Cell lines were maintained at 37°C and 5% CO_2_ and were routinely tested for *Mycoplasma* contamination. To create *Cox10^−/−^Opa1v1*Δ*4* MEFs, we transfected *Opa1v1*Δ*4* MEFs with an all-in-one GeneCRISPR gRNA construct (GeneScript). MEFs expressing active Cas9 with an anchored green fluorescent protein tag were sorted via FACS. After monoclonal selection, the *Cox10* deletion was verified via immunoblot and proteomics analysis. Cell numbers were monitored by Trypan blue exclusion and cell counting using the countess automated cell counter (Thermo Fisher Scientific). Cells were seeded at equal densities and grown to confluency for 72 hours without medium changes unless stated otherwise.

Human and mouse Opa1 was cloned in p3xFLAG CMV10 vector and used to create *Opa1v1*Δ*1*, *Opa1v1*Δ*2*, *Opa1v1*Δ*4*, and *Opa1v1*Δ*10* mutations by site-directed mutagenesis. WT and *Oma1^−/−^* MEFs were transiently transfected with Opa1-Flag for 48 hours, after which cells were treated with 20 μM Carbonyl cyanide m-chlorophenyl hydrazone (CCCP) or DMSO for 2 hours. Cell-free expression was done as in (*81*). Shortly, mouse Oma1 harboring a C-terminal hexahistidine tag together with C-terminally truncated and Myc-tagged human Opa1V1 with *Opa1v1*Δ*2*, *Opa1v1*Δ*4*, and *Opa1v1*Δ*10* mutations was expressed in a bacterial lysate–based, continuous-exchange cell-free expression system using pIVEX2.3d vector in the presence of liposomes. Proteins in Western blot analysis were detected with Myc antibody (CST, no. 2276).

### RNA-seq and qPCR with reverse transcription

Heart tissue was homogenized with Precellys 24 tissue homogenizer (Bertin Instruments) two times for 10 s at 6000 rpm keeping samples on ice in between the cycles. Total RNA was extracted from mouse heart tissue samples using a NucleoSpin RNA kit (Macherey-Nagel), and RNA-seq was performed as in (*50*).

For reverse transcription polymerase chain reaction (PCR), cDNA was synthesized using the GoScript Reverse Transcription Mix (Promega). RNA concentrations were measured with Nanodrop ONE (Thermo Fisher Scientific). Reverse transcription–quantitative PCR (qPCR) was performed using the PowerSYBR Green PCR Master Mix (Applied Biosystems) and QuantStudio 5 and QuantStudio 6 Flex analyzers (Applied Biosystems) with QuantStudio Design & Analysis software (Applied Biosystems). For each independent sample, reverse transcription–qPCR was performed in technical duplicates.

### Imaging

To label mitochondria with mEosEM, 1 μg of the plasmid encoding the protein was introduced into WT and *Opa1v1*Δ*4* MEFs via Gene juice transfection. Mito-mEosEM was a gift from P. Xu (Addgene, plasmid no. 132706). Cells were seeded on live-cell imaging glass bottom dishes (ibidi, μ-dish, 35-mm high). After 48 hours of incubation, mEosEM photoconversion was activated with a 405-nm laser pulse for 3 min (10% laser power). The mitochondrial network (10 μm^2^) was photoconverted, and the activated mitochondrial area after 3 min was set as point zero. The activated signal was followed for 60 min using the excitation lasers at 488 and 561 nm, and the signal area was determined. Normalization to the zero value revealed the percentual area increase. The cells were imaged in DMEM buffered by Hepes at 37°C.

For mitochondrial morphology analysis, WT and *Opa1v1*Δ*4* MEFs were incubated with 20 μM CCCP or DMSO for 2 hours. Mitochondria were immunostained with Atp5ß (Invitrogen, A21351) and labeled with Alexa Fluor 488–conjugated antibody against mouse immunoglobulin G (IgG) (1:500). At least 100 cells from *n* = 3 independent replicates and treatments were analyzed for their mitochondrial length. Mitochondrial populations were classified into tubules, short tubules, and fragmented. Co-localization between mitochondria and ER was analyzed using immunocytochemistry, followed by colocalization analysis (JaCop Analysis, Fiji Plug-In). WT and *Opa1v1*Δ*4* MEFs were seeded on cover slips and harvested 24 hours after seeding. The cells were washed with PBS and fixed with 4% (v/v) paraformaldehyde. After fixation, cells were stained with Tom20 (Proteintech, 66777-1-lg) and Calnexin (Calbiochem, 208880/D19364) primary antibodies. The signals were detected with an Alexa Fluor 488–conjugated antibody against mouse IgG (1:2000) and Alexa Fluor 546–conjugated antibody against rabbit IgG (1:2000).

For confocal imaging, we used the Leica Sp8-X confocal microscope equipped with a 40× oil/numerical aperture (NA) 1.45 objective. For super-resolution analysis, WT and *Opa1v1*Δ*4* MEFs were stained with DMEM supplemented with 250 nM PK mtOrange (GenVivo) for 20 min at 37°C as in (*82*). To remove unbound dye, cells were washed twice with DMEM and imaged 2 hours after staining at 37°C. PK mtOrange was excited at 561-nm wavelength, and stimulated emission depletion microscopy (STED) was performed using a pulsed depletion laser at 775-nm wavelength with 7× line accumulations. Pixel sizes of 20 nm were used for STED nanoscopy and a dwell time of 10 μs. The pinhole was set to 1.0 Airy Units (AU). For image deconvolution, the Huygens software was used. The cells were recorded using a Facility Line microscope (Abberior Instruments) equipped with an Olympus UPLXAPO 60× oil/NA 1.4 objective.

### FACS analysis

Analysis was performed using a Cytek Amnis ImageStreamX Mk II (Cytek Corporation). Cells were selected on the basis of their scatter properties in the bright-field channel; 4′,6-diamidino-2-phenylindole was used for dead cell exclusion, excited by the 355-nm laser; and emission was detected using a 457/45-nm bandpass filter. Only cells with a gradient root mean square value above 55 in channel 1 were recorded. Samples were acquired with a low flow rate at ×40 magnification. Using the IDEAS Image Analysis Software (Cytek Corporation), the features area and diameter were created with an object mask, eroded by 1 pixel {Erode[Object(M01, Ch01, Tight), 1]}.

### Statistics and reproducibility

Sample size was chosen according to our previous experience and common standards. Power analysis was used to predetermine sample size for mouse experiments. The sample size included at least three independent cell culture wells or mice where statistical evaluation was performed. Experiments were repeated as detailed in the figure legends. Error bars represent SD. Mice were assigned to experimental groups on the basis of genotypes available. Analyses were not blinded because experiments were performed and analyzed by the same researcher except for microscopy imaging where samples were numbered. The *n* number for all MEF cell experiments represents independent experimental cell cultures. Data analysis was performed with Prism GraphPad 9 and Instant Clue. Images were processed with ImageJ, and schematics were created with Adobe Illustrator 26.0.2 and BioRender.com. Data that were generated by the German Mouse Clinic were analyzed using R. Tests for genotype effects were made by using Wilcoxon rank sum test or linear models depending on the assumed distribution of the parameter and the questions addressed to the data. A *P* value $ < $0.05 has been used as level of significance; a correction for multiple testing has not been performed.
