# Supplementary material for: Opa1 processing is dispensable in mouse development but is protective in mitochondrial cardiomyopathy
Source: Sci Adv. 2024 Aug 2;10(31):eadp0443. doi: 10.1126/sciadv.adp0443 (PMC11296347; doi:10.1126/sciadv.adp0443)
Supplement: Supplementary file 1 — Figs. S1 to S4 Table S1 Legend for table S2 [file sciadv.adp0443_sm.pdf]

Science Advances
AAAS

Supplementary Materials for

Opa1 processing is dispensable in mouse development but is protective in mitochondrial cardiomyopathy

Sofia Ahola *et al.*

Corresponding author: Thomas Langer, tlanger@age.mpg.de

Sci. Adv. **10**, eadp0443 (2024)
DOI: 10.1126/sciadv.adp0443

The PDF file includes:

Figs. S1 to S4
Table S1
Legend for table S2

Other Supplementary Material for this manuscript includes the following:

Table S2

Supplementary Figure 1

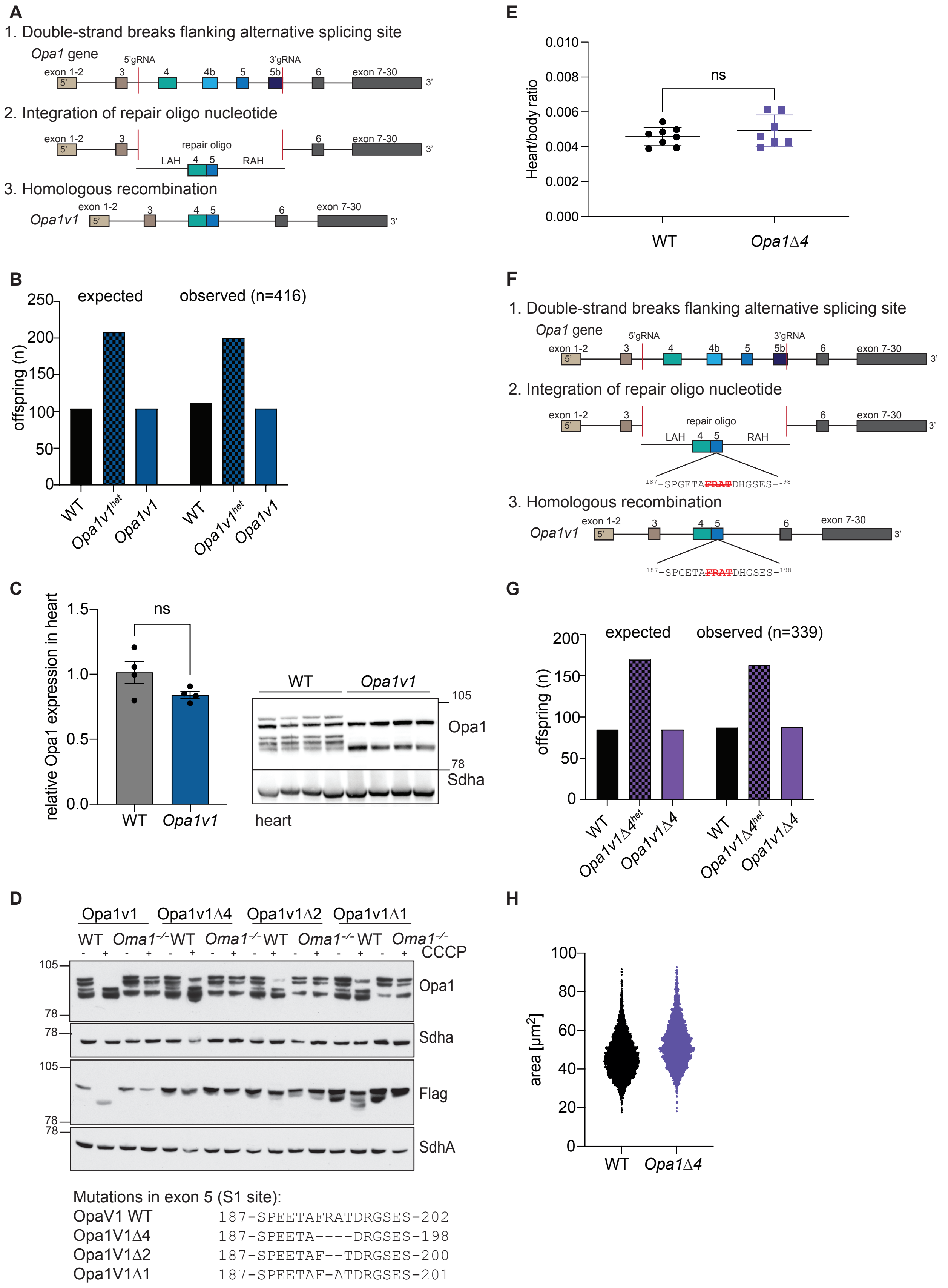

Supplementary Figure 1. Generation of transgenic *Opa1v1* and *Opa1v1Δ4* mice. **(A)** Gene editing strategy for transgenic Opa1v1 mice using CRISPR/Cas9 system. 1. gRNAs flanking the alternative splicing site induced a double-strand break upstream of exon 4 and downstream of exon 5b. The two gRNA templates excise a 10kb region in the Opa1 locus. 2. A defined repair oligonucleotide is annealed to the alternative splicing site by homology arms. 3. The repair oligonucleotide is integrated into the endogenous site by homologous recombination. **(B)** Expected and observed birth ratios of Opa1v1 mice from heterozygous crosses (n=416 pups). **(C)** Quantification of Opa1 levels in the heart of 12-weeks old WT (n=4) and *Opa1v1Δ4* (n=4) mice, unpaired t-test. **(D)** WT and Oma1$^{-/-}$ MEFs transiently expressing Flag-tagged Opa1 variants and treated with CCCP (20 μM) for 2 h. Amino acid sequences at S1 of different Opa1 mutants. **(E)** Heart/body weight ratio from 64-weeks old WT (n=7) and *Opa1v1Δ4* (n=7) mice, unpaired t-test. **(F)** Gene editing strategy for *Opa1v1Δ4* mice showing the deleted four amino acids in the repair oligo in red. **(G)** Expected and observed birth ratios of *Opa1v1* mice from heterozygous crosses (n=339 pups). **(H)** Analysis of the cell size via flow cytometry (Cytek® Corporation). The size of up to 2000 cells in n=3 biological replicates of WT and *Opa1v1Δ4* MEFs was analyzed (in μm2).

Supplementary Figure 2

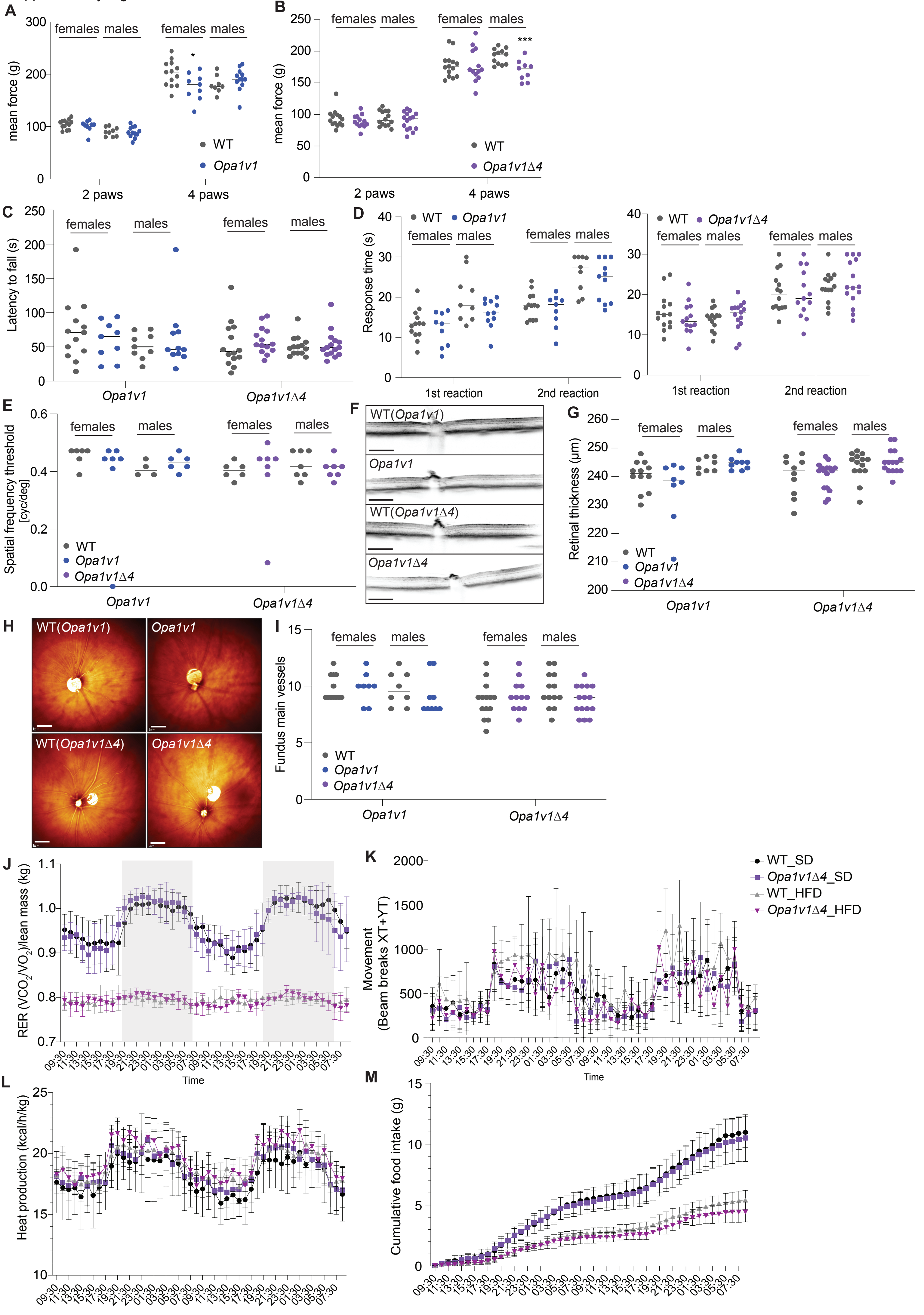

Supplementary Figure 2. Phenotypic analysis of 68-77-weeks-old *Opa1v1* and *Opa1v1Δ4* mice and corresponding WT littermates. (**A**) Grip strength test for *Opa1v1* mice with two and four paws (WT f=13, m=8-9, *Opa1v1* f= 9, m=12). (**B**) Grip strength test for *Opa1v1Δ4* mice with two and four paws (wt f =14, m = 12-14, *Opa1v1Δ4* f=13-14, m= 9-15). **(C)** Accelerating rotarod test for *Opa1v1* and *Opa1v1Δ4* mice (WT f= 13, m= 9, *Opa1v1* f=9, m=11, and WT f=14, m=14, *Opa1v1Δ4* f=14, m=15). **(D)** Hotplate test for *Opa1v1* and *Opa1v1Δ4* mice and corresponding WT littermates showing the time of first and second reaction (WT f=13, m=9, *Opa1v1* f=9, m=11 and WT f=14, m=14, *Opa1v1Δ4* f=13, m=15). (**E**) Virtual drum measurements (WT f=6, m=4, *Opa1v1* f=7, m=6 and WT f=6, m=7, *Opa1v1Δ4* f=7, m=7). (**F**) Representative images of left retinal layers. Scale bar, 1 mm. (**G**) Left retinal thickness (WT f=12, m=8, *Opa1v1* f=8, m=8 and WT f=10, m=14, *Opa1v1Δ4* f=16, m=15). (**H**) Representative images of left fundus. **(I)** Number of blood veins in the left retinal fundus (WT f=12, m=8, *Opa1v1* f=8, m=9 and WT f=14, m=13, *Opa1v1Δ4* f=12, m=15). Scale bar, 1 mm. (**J-M**) Phenomaster data for respiratory exchange ratio (RER), movement, estimated heat production and cumulative food intake of control diet and HFD fed WT and *Opa1v1Δ4* male mice after 10 weeks of diets (WT control diet=7, HFD=4, *Opa1v1Δ4* control diet=6, HFD=3).

Supplementary Figure 3

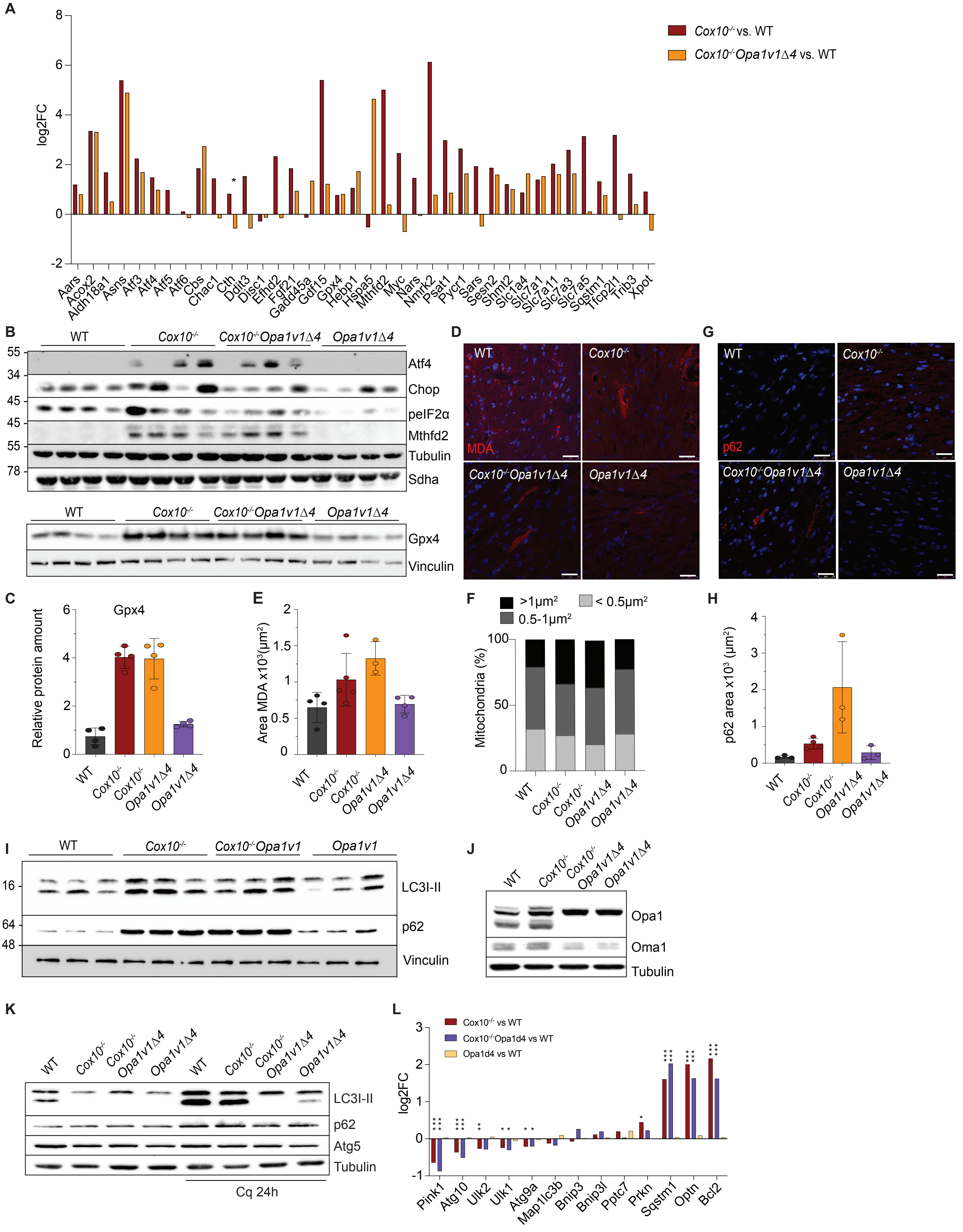

Supplementary Figure 3. Analysis of 2-weeks-old WT, *Cox10*$^{-/-}$, *Cox10*$^{-/-}$*Opa1v1Δ4* and *Opa1v1Δ4* mice and corresponding WT littermates. (**A**) mRNA levels of ISR target genes in hearts of WT, *Cox10*$^{-/-}$ and *Cox10*$^{-/-}$*Opa1v1Δ4* mice, which were determined by Nanostring analysis and which are shown as log 2-fold change between WT and *Cox10*$^{-/-}$ and WT and *Cox10*$^{-/-}$*Opa1v1Δ4.* (**B**) Immunoblot analysis of heart lysates using antibodies against the ISR target protein Mthfd2, peIF2a, the transcription factors Atf4 and Chop, and, in the lower panel, Gpx4. The steady state levels of Sdha and vinculin were monitored for control. (**C**) Quantification of Gpx4 levels in (B). (**D, E**) Representative images for malondialdehyde (MDA) immunohistochemistry staining from the mouse heart with quantification shown in (E) (n=3-4). Scale bars, 25 µm. (**F**) Quantification of mitochondrial sizes in TEM analysis (n=3, >200 mitochondria were analyzed/mouse, data shown as average). (**G, H**) Representative images for p62 immunohistochemistry staining from the mouse heart with quantification shown in (H) (n=3-4). (**I**) Immunoblot analysis of LCI-II and p62 of 2-week-old animals in WT (n=3) , *Cox10*$^{-/-}$ (n=3), *Cox10*$^{-/-}$*Opa1v1* (n=3) and *Opa1v1* (n=3). Lipidated LC3II form and p62 accumulate in *Cox10*$^{-/-}$ and *Cox10*$^{-/-}$*Opa1v1* hearts. (**J**) Immunoblot analysis of Opa1 in MEFs. Scale bars, 25 µm. (**K**) Immunoblot analysis of LC3I-II, p62 and Atg5 in MEFs treated for 24 h with chloroquinone (Cq, 10 µM). (**L**) RNAseq data of autophagy markers in *Cox10*$^{-/-}$ vs WT (red), *Cox10*$^{-/-}$*Opa1v1Δ4* vs WT (purple) and *Opa1v1Δ4* vs WT (yellow), *p< 0.05, **p< 0.01, ***p< 0.001.

Supplementary Figure 4

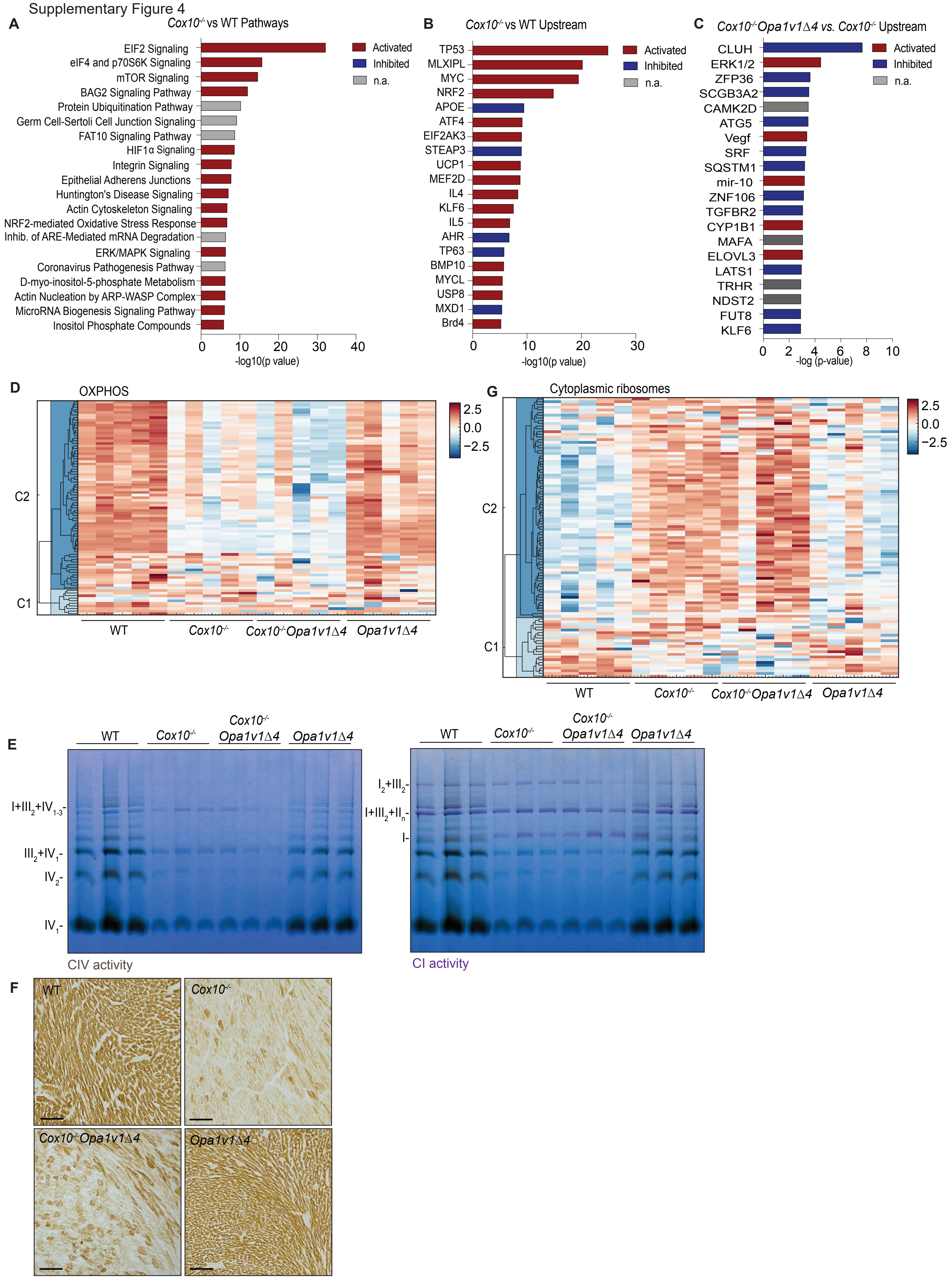

Supplementary Figure 4. Analysis of gene expression and mitochondrial functions of 2-weeks-old WT, *Cox10*$^{-/-}$, *Cox10*$^{-/-}$*Opa1v1Δ4* and *Opa1v1Δ4* mouse hearts. (**A**) Ingenuity pathway analysis of RNAseq datasets for WT and *Cox10*$^{-/-}$ hearts. (**B**) Ingenuity analysis of RNAseq datasets (as in A) to identify predicted upstream regulatory factors whose target gene expression is most significantly altered between WT and *Cox10*$^{-/-}$ hearts. (**C**) Most significantly changed upstream regulatory factors between *Cox10*$^{-/-}$ and *Cox10*$^{-/-}$*Opa1v1Δ4* identified the altered expression of target genes by the Ingenuity analysis. (**D**) Heatmap of Z-scores of OXPHOS-annotated proteins from the heart proteome dataset. (**E**) Blue native gel electrophoresis of mitochondria isolated from the hearts of 2-week-old mice stained for complex IV (CIV) and complex I (CI) activity. (**F**) Representative images for CIV activity staining of mouse hearts of the indicated genotypes. Scale bar, 0.1 mm. (**G**) Heatmap of Z-scores of cytoplasmic ribosome-annotated proteins from the heart proteome dataset.

Supplementary information

Supplementary Table 1: Primers and oligos

Oligo	Sequence
5' gRNA *mOpa1* intron 3/4	AAAACTACAGCACACGGCAA
3' gRNA *mOpa1* exon 5b	CTATGCCCAACAGAAGCGCA
5' gRNA h*OPA1* exon 1	GCGGGATGTGGCGACTACGTCGG
3' gRNA h*OPA1* exon 11	CCTATTTAAAGATAGTTCTC
3' gRNA *mCox10*	GTCGAGGGAATGGTCCACTA
mCox10 sequencing primer forward	GTAATGTGTGCTATTTGTCTGTCTG
mCox10 sequencing primer reverse	ATGTGAGACAATCCACAATCATGT

Oligo	Sequence (5'->3')
Opa1v1 ssODN	TAGAAAAGCCCTGCCCAGCTCAGAAGACCTTGCCAGTTTAG CTCCCGACCTGGACAAGATTACTGAGAGCCTCAGCTTGTTG AAGGACTTCTTCACTGCAGGTTCACCTGGAGAAACAGCATT TCGAGCAACAGATCATGGATCTGAAAGTGACAAGCATTACC GCAAG
Opa1v1Δ4 ssODN	TAGAAAAGCCCTGCCCAGCTCAGAAGACCTTGCCAGTTTAG CTCCCGACCTGGACAAGATTACTGAGAGCCTCAGCTTGTTG AAGGACTTCTTCACTGCAGGTTCACCTGGAGAAACAGCAGA TCATGGATCTGAAAGTGACAAGCATTACCGCAAG

Supplementary Table Legends

Supplementary Table 1. Primers and oligoes used to modify the Opa1 locus by CRISPR-mediated genome editing.

Supplementary Table 2. Protein group DIA-NN quantitative matrix including the log2 LFQ intensity accompanied with protein annotations and statistical analysis (pairwise comparison, one-way ANOVA, and K-Means clustering).
